# Structural Basis for Draxin-Modulated Axon Guidance and Fasciculation by Netrin-1 through DCC

**DOI:** 10.1016/j.neuron.2018.02.010

**Published:** 2018-03-21

**Authors:** Ying Liu, Tuhin Bhowmick, Yiqiong Liu, Xuefan Gao, Haydyn D.T. Mertens, Dmitri I. Svergun, Junyu Xiao, Yan Zhang, Jia-huai Wang, Rob Meijers

**Affiliations:** 1 State Key Laboratory of Membrane Biology, College of Life Sciences, Peking University, Beijing 100871, China; 2 European Molecular Biology Laboratory (EMBL), Hamburg Outstation, Notkestrasse 85, D-22607 Hamburg, Germany; 3 PKU-IDG/McGovern Institute for Brain Research, Peking University, 100871 Beijing, China; 4 State Key Laboratory of Protein and Plant Gene Research, School of Life Science and Peking-Tsinghua Centre for Life Sciences, Peking University, 100871 Beijing, China; 5 Department of Medical Oncology and Department of Cancer Biology, Dana-Farber Cancer Institute, Harvard Medical School, Boston, MA 02115, USA

**Keywords:** Netrin, deleted in colorectal cancer, DCC, Draxin, fasciculation, axon guidance, crystal structure, cysteine knot domain, guidance cue, adhesion

## Abstract

Axon guidance involves the spatiotemporal interplay between guidance cues and membrane-bound cell-surface receptors, present on the growth cone of the axon. Netrin-1 is a prototypical guidance cue that binds to deleted in colorectal cancer (DCC), and it has been proposed that the guidance cue Draxin modulates this interaction. Here, we present structural snapshots of Draxin/DCC and Draxin/Netrin-1 complexes, revealing a triangular relationship that affects Netrin-mediated haptotaxis and fasciculation. Draxin interacts with DCC through the N-terminal four immunoglobulin domains, and Netrin-1 through the EGF-3 domain, in the same region where DCC binds. Netrin-1 and DCC bind to adjacent sites on Draxin, which appears to capture Netrin-1 and tether it to the DCC receptor. We propose the conformational flexibility of the single-pass membrane receptor DCC is used to promote fasciculation and regulate axon guidance through concerted Netrin-1/Draxin binding.

**Video Abstract:**

## Highlights

• Crystal structure of cysteine knot domain of Draxin in complex with DCC
• Crystal structure of Netrin-1 in complex with a Draxin fragment
• Netrin-1 contains a competing binding site for DCC and Draxin on the EGF-3 domain
• Draxin tethers Netrin-1 and DCC together to promote fasciculation

## Teaser

Liu et al. report through structural investigations how Draxin associates both with Netrin-1 and its cognate receptor DCC to mediate axon guidance and fasciculation.

## Introduction

The wiring of commissural neurons in the developing spinal cord is central to the development of bilateral symmetry. Commissural neurons extend axons dorsoventrally in the spinal cord that eventually cross over the midline to establish this symmetry (Chédotal, 2014). During this process, axon guidance cues, particularly the prototypical cue molecule Netrin-1, direct the growth cone situated at the tip of the axon from the roof plate toward the floor plate. Once it reaches the floor plate, the axon turns to cross the midline. Recent data appear to demonstrate that Netrin-1 derived from neural progenitors within the ventricular zone provides an adhesive axon growth substrate to guide axons through haptotaxis, and to promote axon fasciculation (Dominici et al., 2017, Varadarajan et al., 2017). Structural investigations have identified three separate receptor-binding sites on the Netrin-1 molecule at its N-terminal laminin and three following EGF domains (Finci et al., 2014, Grandin et al., 2016, Xu et al., 2014). These sites are used as a platform for Netrin-1 to engage several receptors to trigger a diverse set of signals that can determine axon navigation trajectory or cell fate. When Netrin-1 binds two DCC receptors, the homo-dimerization process has been linked to chemo-attraction of an axon (Finci et al., 2014, Kennedy et al., 1994). When DCC is co-expressed with UNC5, the Netrin-1-mediated hetero-dimerization of DCC-UNC5 turns the axon response to the opposite effect, namely chemo-repulsion (Finci et al., 2014). Furthermore, it has been shown that when a migrating cell is depleted of Netrin-1, and DCC clustering is prevented, apoptosis might ensue (Grandin et al., 2016, Krimpenfort et al., 2012, Mehlen et al., 1998).

The guidance cue Draxin (dorsal repulsive axon guidance protein) was characterized as a repulsive cue that prevents axons from misprojecting before midline crossing (Islam et al., 2009). Draxin knockout mice showed defects in fasciculation as well, indicating Draxin has an effect on axonal adhesion between pioneering and follower axons. In this context, Draxin was shown to interact with DCC present on the growth cone of the axon (Ahmed et al., 2011, Meli et al., 2015). Interestingly, a high-throughput screen for axon guidance cues and receptors present at the ventral midline revealed that Netrin-1 and Draxin bind directly to each other as well (Gao et al., 2015).

**Figure 1:**
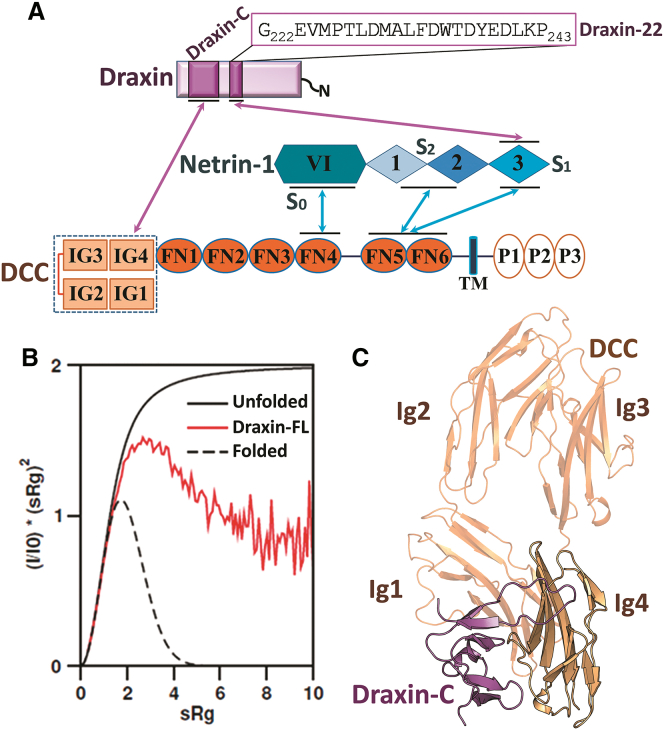
Overall Description of Draxin, DCC, and Netrin-1 Interactions (A) Schematic of the interactions between the soluble guidance cues Draxin, Netrin-1, and the DCC receptor ectodomain. Draxin interacts with DCC through the C-terminal Draxin-C domain with the Ig4 domain of DCC (pink arrow). Draxin interacts with Netrin-1 through a flexible region lying next to Draxin-C, with the EGF-3 domain of Netrin-1 (pink arrow). DCC interacts with Netrin-1 through three sites (blue arrows). (B) Dimensionless Kratky plot of the SAXS data demonstrating the partially folded nature of hDraxin in solution. Theoretical Kratky representations for compact folded (dotted black line), fully unfolded (solid black line), and the experimentally derived full-length human Draxin (solid red line) are shown. (C) Ribbon representation of the structure of rDraxin-C in complex with DCC^Ig1Ig4^. rDraxin-C is shown in magenta. It mainly interacts with DCC-Ig4 (beige).

To date, it is not clear how Netrin-1 and Draxin coordinate to affect axon fasciculation and guidance. Cell binding assays indicated Draxin binds DCC at the N-terminal, membrane-distal region containing four immunoglobulin (Ig) domains (Ahmed et al., 2011). These domains form a horseshoe-shaped platform, which is common among neuronal Ig superfamily receptors (Chen et al., 2013). In contrast, Netrin-1 binds the DCC receptor on the membrane-proximal fibronectin (FN) domains FN4 and FN5-FN6, separate from where Draxin binding occurs (Figure 1A). Here, we present a structural characterization of Draxin itself and crystal structures of Draxin in complex with a fragment of DCC as well as with Netrin-1. We show that Draxin is largely unstructured, but that it uses a small C-terminal domain (Draxin-C) to bind DCC N-terminal Ig domains and an upstream conserved peptide motif (Draxin-22) to bind Netrin-1. Together with complementary biophysical experiments, we untangle the interactions between Draxin, Netrin-1, and DCC. We propose that Draxin facilitates Netrin-1 to act as a hub for receptors that switch between *cis* interactions involving the same axon, and *trans* interactions involving other axons or cellular substrates, to facilitate axonal adhesion and fasciculation.

## Results

### Draxin Contains a Small Cysteine Knot Domain that Binds DCC

Draxin is predicted to consist of a signal peptide for secretion, an unstructured region covering residues 25 to 245, and a C-terminal domain (Figures 1A, S1, and S2). The folding properties of freshly purified human Draxin (hDraxin) expressed in HEK293T cells were tested by small angle X-ray scattering (SAXS; Figure 1B). The derived SAXS parameters suggest that in solution hDraxin is predominantly monomeric (Table S1). Ensemble analysis of the SAXS data (Figure S3A) shows a bi-modal size distribution, indicating that folded and unfolded states of the N-terminal part of hDraxin co-exist in solution. To identify which region of Draxin interacts with DCC, we attempted co-crystallization between full-length rat Draxin (rDraxin) and a fragment of the rat DCC receptor consisting of Ig domains 1 to 4 (rDCC^Ig1-Ig4^), since Draxin binding was found to involve the N-terminal four domains of DCC (Ahmed et al., 2011).

A crystal structure was determined by molecular replacement using the known DCC^Ig1-Ig4^ structure at 2.5 Å resolution (Figures 1C, S4A, and S4B). In the refined structure, a fragment of rDraxin consisting of the C-terminal region that extends from Gly264 to Pro329 can be built. The electron density for this rDraxin-C domain is continuous for residues Gly264 to Ala311 and Arg317 to Pro329 with weak density linking Ala311 to Arg317. All residues that form an interface with the DCC molecule are well defined in the electron density. When the crystals are dissolved and analyzed by SDS-PAGE, it appears the full-length rDraxin molecule is present in the crystal (Figure S3B). The solvent content for a full-length rDraxin/DCCIg1-Ig4 co-crystal is 60%, which gives enough space to accommodate the large portion of the disordered remainder of the rDraxin molecule, which is not visible in the electron density map.

**Figure 2:**
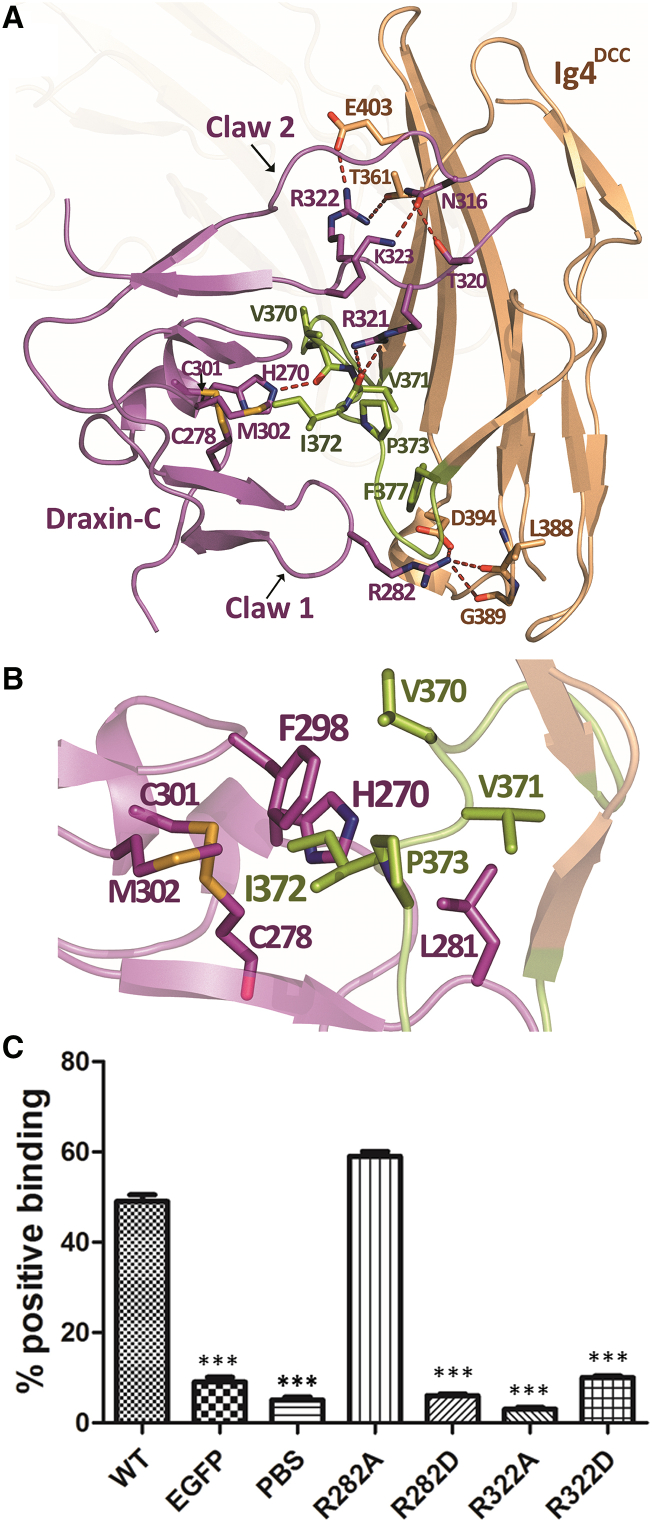
Characterization of Draxin/DCC Binding (A) Overview of the interactions between rDraxin-C and the Ig4 domain of rDCC^Ig1Ig4^. Residues mediating important interactions between the two proteins are shown as sticks and labeled. Salt bridge and hydrogen bond interactions are shown as dashed lines. The CD loop on the Ig4 domain of DCC is colored in green. (B) Detailed view of the hydrophobic hotspot of the rDraxin-C/r DCC^Ig1Ig4^ complex. Residue Ile372 from the CD loop (colored in lemon) of rDCC-Ig4 stacks between His270 and Phe298 of rDraxin-C to form the core of the hydrophobic cluster. The core is surrounded by the hydrophobic side chains of Val370, Val371, and Pro372 from the CD loop of DCC^Ig1Ig4^ and Cys278, Leu281, Cys301, and Met302 from rDraxin-C. The view is rotated 45 degrees along the vertical axis with regard to (A). (C) Cell-binding assays for full-length wild-type (WT) rDCC showing the percentage of DCC presenting HEK293Tcells that bind rDraxin WT and mutants Arg282Ala, Arg282Asp, Arg322Ala, and Arg322Asp. As a control, eGFP and PBS were used. Data represent mean ± SE (n = 100 for each group). One-way ANOVA, followed by a post hoc Scheffé’s test, was performed. ^∗∗^p < 0.001 compared with WT.

In the structure of the rDraxin-C/DCC^Ig1-Ig4^ complex, rDraxin-C essentially binds to the Ig4 domain of DCC. Two loops extend out from rDraxin-C, clamping at the CD loop of the Ig4 domain of DCC like a lobster grabbing her prey (Figure 2). The rDraxin-C structure consists of two sub-domains we have designated Claw1 (residues Gly264 to Asn290) and Claw2 (residues Arg291 to Pro329) of the lobster, which are kept together by a disulfide bond between Cys278 and Cys301. Each subdomain contains two finger-shaped loops that are kept together by disulfide bonds, a configuration that is typical for a cysteine knot domain. The topology of rDraxin-C is similar to the C-terminal region of the Dickkopf (DKK) protein that is involved in Wnt signaling (Cheng et al., 2011, Mao et al., 2001). Superposition of the rDraxin-C structure with DKK shows that the overall domain architectures are quite similar (Figure S3C). The disulfide bond pattern in the rDraxin-C and DKK structures is identical (Figure S3D).

When the two claws of rDraxin-C pinch the rDCC^Ig4^ domain around the CD loop (Figure 2A), Claw1 clamps the CD loop at the bottom of rDCC^Ig4^, whereas Claw2, a much larger loop, reaches the top of rDCC^Ig4^. A total of eleven hydrogen bonds, largely arginine mediated, are formed between rDraxin-C and rDCC^Ig1-Ig4^, which warrant binding specificity (Lo Conte et al., 1999). Notably, the only contacts between rDraxin-C and the rDCC^Ig1-Ig4^ horseshoe outside of the Ig4 domain are two hydrogen bonds from rDraxin-C to the Ig1 domain of rDCC^Ig1-Ig4^. There are also extensive hydrophobic interactions between the two binding partners to ensure binding affinity (Figure 2B). In particular, Ile372^DCC^ is the most significant one, protruding out from this CD loop to be surrounded (anti-clockwise in Figure 2B) by Phe298^Draxin^, Met302^Draxin^, the disulfide pair Cys278^Draxin^-Cys301^Draxin^, His270^Draxin^, and Leu281^Draxin^, which might be the energetic “hot spot.”

To verify the contributions of the individual residues in the binding interface between rDCC^Ig1-Ig4^ and rDraxin-C, we performed mutagenesis on full-length rDraxin and tested binding to COS cells expressing full-length wild-type DCC on the cell surface (Figure 2C). The residue Arg282^Draxin^ situated on Claw1 seems to be involved in several interactions with DCC^Ig4^. A charge-reversal mutant (Arg282Asp) abolishes binding of rDraxin to DCC, whereas an Arg282Ala mutant seems to have little effect (Figure 2C). Mutants of residue Arg322^Draxin^ present on Claw2 of rDraxin were also tested for binding to DCC presented on COS cells. Replacement of the side chain of Arg322^Draxin^ with alanine or aspartate leads to a drastic reduction in binding.

### Crystal Structure of a Human Draxin/Netrin-1 Complex

**Figure 3:**
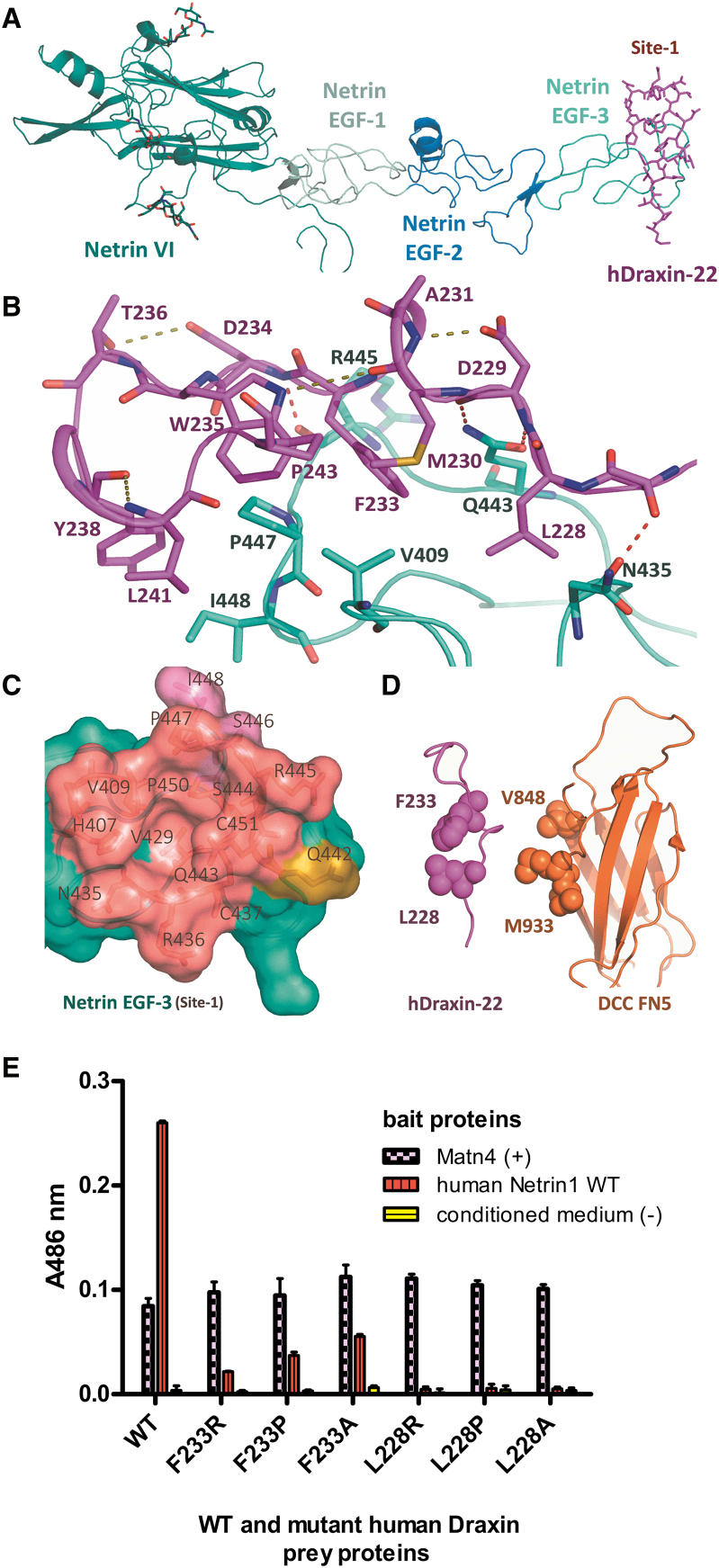
Crystal Structure of the hNetrin-1/hDraxin-22 Peptide (A) An overview of the complex showing hDraxin-22 peptide (in magenta) represented as sticks binding to the Netrin-1 on the EGF-3 domain, represented in ribbon. Netrin-1 consists of the N-terminal laminin VI domain (in deep teal), followed by three EGF domains (in pale cyan, marine, and cyan). (B) Important residues involved in hNetrin-1/hDraxin-22 interaction interface: residues Thr227, Asp229, and Asp234 of hDraxin-22 form hydrogen bonds with Asn435, Gln443, and Arg445 of hNetrin-1, respectively, while Leu228 and Phe233 of hDraxin-22 are involved in hydrophobic interactions. The hydrogen bonds between hDraxin-22 and Netrin-1 are shown in red, while intra-Draxin hydrogen bonds are shown in deep olive. (C) A comparison between the residues of Netrin-1 involved in Draxin and DCC binding using a surface representation of Netrin-1 site 1 located at the EGF3 domain (in cyan). The residues interacting only with Draxin are colored in pink, only with DCC are colored in olive, and with both Draxin and DCC are colored in deep salmon. These residues form a hydrophobic cavity at site 1, surrounding residue Val429 of Netrin. (D) Comparison of the crucial residues involved in Netrin-1 binding for Draxin (Leu228 and Phe233) and DCC (Met933 and Val848). (E) AVEXIS binding results for Netrin/WT and mutant Draxin interactions. Human Netrin-1 WT bait protein (VI+V) interacts with Draxin full-length prey proteins with indicated mutations. Matn-4 bait used as the internal positive control; conditioned medium as the negative control. The A486 nm values correspond to the average of three repeats, error bars represent mean ± SD, and results were confirmed in two independent experiments.

Just upstream of the rDraxin-C domain (Figure S1), there is a conserved region of 22 amino acids that interacts with Netrin-1 (Figure 1A; Gao et al., 2015). A crystal structure was determined of a fragment of human Netrin-1 (hNetrin-1) consisting of the laminin and three EGF domains (residues 22 to 455) in complex with a human Draxin peptide with a length of 22 amino acids (hDraxin-22) corresponding to the region from Gly222 to Pro243 (Figures 3A and S4C).

In the hDraxin/hNetrin-1 complex, the hDraxin-22 peptide is bound to the EGF-3 domain of Netrin-1 in an extended hook-like fashion, perpendicular to the long axis of the rigid rod-like shape of the Netrin-1 molecule. The key feature of the hDraxin/hNetrin-1 complex structure is that there are two cavities on the EGF-3 domain of hNetrin-1 that are filled by the hydrophobic side chains of residues Leu228^Draxin^ and Phe233^Draxin^ of the hDraxin-22 peptide (Figure 3B). These residues are positioned to point into the cavity and are stabilized by a network of specific hydrogen bonds between the hDraxin-22 peptide and hNetrin-1 residues as well as intra-Draxin hydrogen bonds.

Previously, it was shown that Draxin interferes with Netrin-1 binding to DCC, and that this effect is localized in the 22 aa Draxin fragment (Gao et al., 2015). The structure reveals that the hDraxin-22 peptide binds hNetrin-1 at the same location as the FN5 domain of DCC. The binding epitopes overlap to a large extent (Figure 3C), surrounding the two cavities formed on the EGF-3 domain of Netrin-1. The FN5 domain of DCC employs β strands A and G to position Val848^DCC^ and Met933^DCC^ to fill the EGF-3 domain cavities of Netrin-1 (Finci et al., 2014, Xu et al., 2014). The hDraxin-22 peptide uses a hydrogen bonding network to align Phe233^Draxin^ in the same position as Val848^DCC^ from DCC, and Leu228^Draxin^ in the same position as Met933^DCC^. It is interesting to note that in the Netrin-1/DCC structure, the Netrin-1 residue Q433^netrin^ forms two hydrogen bonds to the main chain of DCC, facilitating the hotspot binding to Val848^DCC^ and Met933^DCC^ (Finci et al., 2014). In the structure of hNetrin-1/hDraxin-22, the same Q433^netrin^ forms two hydrogen bonds to the main chain of Draxin to orient Phe233^Draxin^ and Leu228^Draxin^ for binding. The buried surface area is very similar between the Draxin/Netrin-1 interface (605 Å^2^) and the Netrin-1/DCC interface (609 Å^2^). Similarly, the surface complementarity values between the Draxin/Netrin-1 interface (0.67) and the Netrin-1/DCC interface (0.66) are comparable. Since there does not seem to occur any refolding of the hDraxin-22 peptide upon binding to Netrin-1, we speculate that solvation-related energy effects are the main determinant in the relatively strong binding of Draxin to Netrin-1 (Kd = 10 nM; Gao et al., 2015) compared to DCC (estimated Kd for the Fn5 domain alone is 5 μM; Xu et al., 2014).

### Binding Assays Confirm Draxin Binding to Netrin-1 Occurs Only through the EGF-3 Domain

To validate the contribution of the individual Draxin residues to Netrin-1 binding, AVEXIS assays were performed (Bushell et al., 2008) with full-length hDraxin (Gao et al., 2015). Based on the crystal structure presented here, point mutations were introduced in the region of Draxin that interacts with Netrin-1. Mutagenesis of Phe233^Draxin^ to alanine leads to a reduction in Netrin-1 binding, whereas a Phe233Arg^Draxin^ mutant completely abolishes binding (Figure 3E). Importantly, mutagenesis of Leu228^Draxin^ has an even stronger effect because the Leu228Ala^Draxin^ mutant is sufficient to disrupt interaction with Netrin-1. This confirms that the residue Leu228^Draxin^, which fills the pocket at the EGF3 domain of Netrin-1, is most crucial for binding, just like Met933^DCC^, which occupies the same pocket in the Netrin-1/DCC interaction. The observation that single point mutants within the region of Draxin identified to bind the EGF-3 domain of Netrin-1 in the crystal structure can disrupt binding between Draxin and Netrin-1 confirms that this is the only mode of interaction between these guidance cues, despite the intrinsically disordered properties of Draxin.

## Discussion

**Figure 4:**
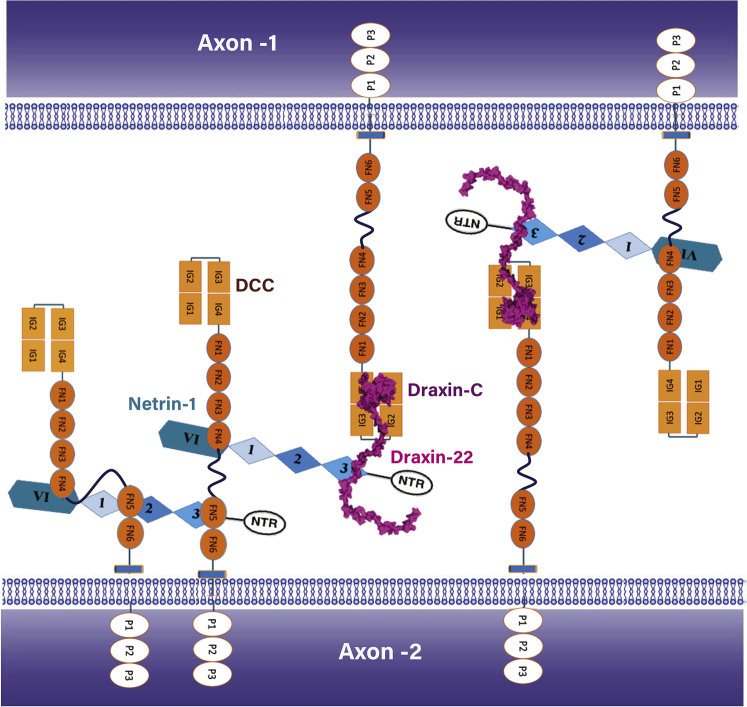
Proposed Model for hNetrin-1- and Draxin-Mediated Adhesion between Axons through DCC Two opposing axons decorated with DCC (orange, individual Ig domains depicted as ovals and fibronectin domains as boxes). hNetrin-1 (different shades of blue) binds DCC at the laminin (VI) domain as well as the EGF domains (sites 1 and 2). Draxin (purple) competes with DCC to bind hNetrin-1 at site 1 and crosslinks hNetrin-1 with a DCC molecule on the opposite axon.

The crossing of the midline by commissural axons in the developing spinal cord of vertebrae involves an intricate combination of guidance cues, including Draxin and Netrin-1 (Dudanova and Klein, 2013). Although Draxin was identified as a repulsive guidance cue based on its *in vitro* activities, the main phenotype observed in Draxin knockout mice is defasciculation. Draxin seems to facilitate the bundling of axons together, and its absence leads to the straying of individual commissural axons. Here we provide structural evidence that Draxin interacts both with Netrin-1 and DCC. Draxin binds to the horseshoe-shaped N-terminal Ig domains of DCC, distant from the cell membrane. The crystal structure of the C-terminal region of Draxin in complex with rDCC^Ig1-Ig4^ reveals a relatively weak binding site, which is verified with structure-derived mutants of Draxin in cell-based binding studies. Draxin also contains a Netrin-1 binding site, just 20 amino acids N-terminal to Draxin-C, the DCC binding domain. It covers a 22-residue region that is evolutionary conserved, but is intrinsically unstructured. The crystal structure of hNetrin-1 in complex with an hDraxin-22 peptide illustrates how Draxin binds to Netrin-1. Strikingly, Draxin binds to Netrin-1 on the EGF-3 domain of Netrin-1, involving the same region that constitutes binding site 1 for DCC. Draxin outcompetes DCC for binding to Netrin-1 on site 1, based on kinetics experiments (Gao et al., 2015, Xu et al., 2014). From the binding configurations between Draxin, DCC, and Netrin-1, it is possible to suggest a model for how Draxin promotes axon fasciculation (Figure 4). The close proximity of the DCC and Netrin-1 binding sites on Draxin enables Draxin to capture Netrin-1 molecules on the tip of DCC, away from the cell membrane of the axon. The relatively strong Draxin/Netrin-1 complex can build a bridge between two axons decorated with DCC, initiating adhesion and therefore fasciculation between the axons.

The classical model for Netrin/DCC clustering suggests that two DCC receptor ectodomains are paired by a Netrin-1 molecule, triggering the dimerization of the cytosolic domains of DCC (Finci et al., 2014, Stein et al., 2001). This dimerization will then lead to the formation of a supramolecular complex around the cytosolic domains of DCC (Lai Wing Sun et al., 2011). Surprisingly, structural studies on Netrin/DCC revealed three separate binding sites for DCC on Netrin-1 (Finci et al., 2015, Xu et al., 2014), which indicates that the pairing of DCC and other Netrin receptors is more complex. One of the binding sites (site 2) can alternatively bind DCC and UNC5, which leads to a switch from chemo-attraction to chemo-repulsion (Finci et al., 2015, Grandin et al., 2016). Now we show that another binding site (site 1) can alternatively bind DCC and Draxin. The multivalent interactions between Netrin-1 and DCC, modulated by other receptors and soluble factors such as Draxin, may be involved in the movement of the axon along a substrate-bound Netrin-1 gradient (Dominici et al., 2017, Varadarajan et al., 2017), implying that axon guidance is a more complex process than was previously thought. The exploration of the mechanistic principals underlying this key neuronal developmental process seems to enter an exciting phase.

The structural details presented here may provide general insights into the mode of action of neuronal receptors containing Ig and fibronectin domains that act as beads on a string. For instance, it was recently shown that DSCAM interacts with guidance cues Slit and Netrin-1 (Dascenco et al., 2015, Liu et al., 2009). Similar to Draxin/DCC, Slit binds to the horseshoe-shaped N-terminal region of DSCAM (Dascenco et al., 2015). As these neuronal receptors show great conformational flexibility, they allow different guidance cues that are bound to different regions of the receptor to engage each other. This may lead to an intricate network of interactions that are concentration dependent in a spatiotemporal fashion, governed by the gradients of the guidance cues. In parallel, associate guidance cues like Draxin and Dickkopf may act as recruiters that link different guidance cue systems together. In fact, Draxin has been shown to bind to LRP6, a receptor in the canonical Wnt pathway (Miyake et al., 2009). Draxin could therefore link Netrin-1-mediated dorsal-ventral axon guidance with Wnt-mediated anterior-posterior axon guidance. Further investigations into these molecular handshakes will reveal further complexities in axon guidance and cell migration.

## STAR★Methods

### Key Resources Table

**Table:** 

REAGENT or RESOURCE	SOURCE	IDENTIFIER
**Antibodies**		

penta-His primary antibody	QIAGEN	cat#34660; RRID: AB_2619735
Horse radish peroxidase-linked secondary antibody	Thermo Fisher Scientific	cat# 32230; RRID: AB_1965958
FLAG monoclonal antibody	Cell Signaling	3916S; RRID: AB_10694611
Mouse monoclonal anti rat CD4 (clone OX-68) (for AVEXIS prey and bait protein normalization)	Bio-Rad/AbD Serotec	MCA1022R; RRID: AB_567282

**Chemicals, Peptides, and Recombinant Proteins**		

Draxin peptide: GEVMPTLDMALFDWTDYEDLKP	Genscript	hDraxin-22
Nitrocefin CAS 41906-86-9	Merck	Cat# 484400

**Deposited Data**		

Crystal Structure of rDraxin/ rDCC^Ig1-Ig4^ complex	This study	PDB: 5Z5K
Crystal structure of hNetrin-1/hDraxin-22 complex	This study	PDB: 6FKQ
SAXS experimental data	This study	SASBDB: SASDBZ6

**Experimental Models: Cell Lines**		

HEK293T cells	ATCC	CRL-11268
COS Cells	ATCC	CRL-1651
FreeStyle 293-F Cells	Thermo Fisher Scientific	R79007

**Oligonucleotides**		

5′ ATTTA GCGGCCGCC ATGGCAGGGTCCGTC 3′	This study	rat Draxin forward primer signal sequence.
5′ GCCCC GAGCTC GATGTTGATGAAAGATCCC 3′	This study	rat Draxin, reverse primer
5′ TGGAGCCACCCCCAGTTCGAGAAGGGCGGCT CTCTTCATTTTGTGTCTGAACC 3′	This study	rat DCC (residues 39-422) forward primer
5′ TTAAA GGTACC GGCGGCTGGAGCCACCCC CAG 3′	This study	rat DCC reverse primer
5′ AATAT GAGCTC GATGGCAGGCTTGGGG 3′	This study	rat DCC reverse primer
5′ GCGGCCGCCACC ATGGCTGGGCCTGCCATC CACACCGCTC 3′	(Gao et al., 2015)	human Draxin forward primer with signal sequence
5′ GGCGCGCC GACGTTGATGAAGGATCCCT GGTC 3′	(Gao et al., 2015)	human Draxin full length reverse primer
5′ ATAAGAAT GCGGCCGC CATGGGAACCCTC 3′	This study	common primer from pXLG to AVEXIS vector, forward primer
5′ A GGCGCGCC ATGGTGGTGGTGGTGGTG GAGCTC 3′	This study	common primer from pXLG to AVEXIS vector, reverse primer

**Recombinant DNA**		

Plasmid: pXLG-rDraxin	This study	N/A
Plasmid: pXLG-hNetrin-1	(Finci et al., 2014)	N/A
Plasmid: rDCC ^Ig1-Ig4^	(Chen et al., 2013)	N/A
Plasmid: AVEXIS-hDraxin-prey	(Gao et al., 2015)	Addgene #36148
Plasmid: AVEXIS-hDraxin-bait	(Gao et al., 2015)	Addgene #36149

**Software and Algorithms**		

HKL2000	(Otwinowski and Minor, 1997)	N/A
Phaser	(Bunkóczi et al., 2013)	N/A
Coot	(Emsley et al., 2010)	N/A
Phenix	(Afonine et al., 2012)	N/A
Molprobity	(Chen et al., 2010)	N/A
Pymol	The PyMOL Molecular Graphics System, Version 1.7.x Schrödinger, LLC	N/A
ESPRIPT	(Robert and Gouet, 2014)	N/A
MOSFLM	(Battye et al., 2011)	N/A
SCALA	(Evans, 2006)	N/A
MOLREP	(Vagin and Teplyakov, 2010)	N/A
REFMAC5	(Murshudov et al., 2011)	N/A
CCP4 Suite	(Winn et al., 2011)	N/A
Prism	GraphPad	N/A
RADAVER	Franke et al. (2012)	https://www.embl-hamburg.de/biosaxs/download.html
PRIMUS/Qt	Petoukhov et al. (2012)	https://www.embl-hamburg.de/biosaxs/download.html
CRYSOL	Petoukhov et al. (2012)	https://www.embl-hamburg.de/biosaxs/download.html
DAMMIF	Franke and Svergun, 2009	https://www.embl-hamburg.de/biosaxs/download.html
SASVIEW	NSF DANSE	http://www.sasview.org

**Other**		

Mutagenesis kit	Agilent Techologies	#200521
Dulbecco’s modified eagle medium	DMEM, Biochrom	cat# F 0435
L-glutamine	Biochrom	cat# K 0293
fetal-bovine serum	Biochrom	cat# S 0615
Protease Inhibitor Cocktail tablets	cOmplete ULTRA Tablets, Roche	05892791001
Ni-Sepharose Excel resin	GE Healthcare	cat# 17-3712-02
HiLoad 16/60 Superdex 75 column	GE Healthcare	#28-9893-33
Nickel-NTA agarose beads	QIAGEN	Cat No./ID: 30250

### Contact for Reagent and Resource Sharing

Further information and requests for reagents should be directed to and will be fulfilled by the Lead Contact, Rob Meijers (r.meijers@embl-hamburg.de).

### Method Details

#### Protein production and purification

The DNA fragment of full-length rDraxin was amplified from rat embryonic cDNA library, and subcloned into the pXLG vector kindly provided by David Hacker and Florian Wurm (Protein Expression Core Facility, EPFL Lausanne, Switzerland). The rat rDCC ^Ig1-Ig4^ construct previously described (Chen et al., 2013) was subcloned into the pXLG vector covering residues 39 to 421. The DNA fragment of full-length human Draxin (aa 1-349, GenBank: KM655686) was amplified and subcloned into modified AVEXIS bait vector (Addgene plasmid #36148) with C-terminal removal of CD4 and replaced by a purification tag (TEV-StrepII-linker-StrepII-linker-9xHis) for use in SAXS. Human Netrin-1 covering residues 39 to 457 was subcloned into pXLG as previously described (Finci et al., 2014). For rDCC ^Ig1-Ig4^ and hNetrin-1, the PSG1 signal peptide was used to secrete the protein and all constructs contained a C-terminal 6xHis tag. The proteins were expressed in adherent HEK293T cells (ATCC Catalog No. CRL-11268) through transient transfection in roller bottles. Cells were cultured in Dulbecco’s modified eagle medium (DMEM, Biochrom cat# F 0435), containing nonessential amino acids, 2 mM L-glutamine and 0.2% fetal-bovine serum (Biochrom, cat# K 0283,cat# K 0293, cat# S 0615) at 37°C in a 5% CO_2_ atmosphere in a Wheaton incubator. The expressed protein was secreted into the culture medium, which was harvested four to six days post transfection. The medium was filtered through filter paper (Whatman). Expression was confirmed by western blot analysis using a penta-His primary antibody (QIAGEN, cat#34660) and a HRP-linked secondary antibody (Pierce, cat# 32230). Protease Inhibitor Cocktail tablets [cOmplete ULTRA Tablets, Mini, EDTA-free, EASYpack (05892791001 Roche)] were added to the medium (2 tablets per liter) immediately after harvesting.

Both rDraxin and rDCC ^Ig1-Ig4^ containing media were dialyzed in 20 mMTris-HCl, pH7.5, 200mMNaCl. These proteins were affinity purified by Ni column (QIAGEN Ni-NTA agarose), followed by size exclusion chromatography using a HiLoad 16/60 Superdex 200 column from GE Healthcare. Harvested media containing recombinant hNetrin-1 was then incubated at 4°C with 1.5 ml Ni-Sepharose Excel resin (GE Healthcare, cat# 17-3712-02) per liter medium, overnight with slow stirring. hNetrin-1 was eluted in 1ml fractions with 20 mM sodium phosphate, 500 mM NaCl, 500 mM imidazole at pH 7.4. The total elution volume collected was 5ml from 2 l of conditioned media. Next, the eluent from the affinity chromatography was subjected to size exclusion chromatography using a HiLoad 16/60 Superdex 75 prep grade column equilibrated in 50 mM MES buffer pH 6.0, containing 250 mM NaCl and 1 mM DTT, with a flow rate of 1 mL per minute. The hNetrin-1 peak from the size exclusion step was collected in 0.5 mL fractions in a deep well block. After inspection through SDS-PAGE gel, fractions containing hNetrin-1 with > 90% purity were combined and concentrated using an Amicon Ultra-4 Centrifugal Filter (Millipore, cat# UFC800324) to 1mg/ml (∼20 uM).

#### Crystallization and structure determination

Purified rDraxin and rDCC^Ig1-Ig4^ were combined in 1.2:1 molar ratio, and incubated at 4**°**C for 2 hours before loading onto the size exclusion column. The complex peak fractions were collected and concentrated to ∼12mg/ml for crystallization. Crystals appeared readily at a few conditions, the best of which is in the No.8 condition of PEG/Ion Screen (HR2-126 Hampton). It was optimized to 0.2 M KCl, 3% PEG 3350 to get well-diffracting crystals. Diffraction data were collected at SSRF (Shanghai Synchrotron Radiation Facility, China) beamline BL17U and processed with HKL2000 (HKL Research) (Otwinowski and Minor, 1997). The structure was determined by molecular replacement with Phaser (Bunkóczi et al., 2013) using the DCC^Ig1-Ig4^ structure (PDB: 3LAF) as the search model. The structure was built in Coot (Emsley et al., 2010) and refined using Phenix (Afonine et al., 2012), with 5% randomly selected reflections used for cross-validation. The final working R and free R factors were 20.7% and 24.7%, respectively (Table S2). The stereochemistry was checked with Molprobity (Chen et al., 2010), indicating good overall geometry with only 0.4% of the residues in disallowed regions of the Ramachandran plot. Structure diagrams were prepared with Pymol (The PyMOL Molecular Graphics System, Version 1.7.x Schrödinger, LLC). Sequence alignments were prepared with ESPRIPT (Robert and Gouet, 2014).

A 22-mer peptide (sequence: GEVMPTLDMALFDWTDYEDLKP) corresponding to the residue spanning 222-243 of human Draxin (UniProtKB entry: Q8NBI3 DRAXI_HUMAN) was ordered from Genscript. The purity of the synthesized peptide was > 75%. A peptide stock of 1mM was prepared by dissolving the peptide in water, first by lowering the pH to 5.5 and then gradually bringing it back to neutrality.

hNetrin-1 and hDraxin-22 peptide were mixed at 1:1.5 molar ratio and incubated for an hour prior to concentration to 6.5 mg/mL. Crystals were obtained in two conditions containing 1.6 M ammonium sulfate and 0.1 M Sodium citrate, at pH 4 and pH 5, respectively. Crystals were cryo-protected in 0.1M Sodium citrate (pH 4.0 or pH 5.0, based on the crystallization condition), 1.6M ammonium sulfate, 15% (v/v) Ethylene glycol prior to flash-cooling to 100K. X-ray diffraction data were collected on the P14 beamline of EMBL Hamburg situated at the PETRA3 synchrotron. The beamline was equipped with a Pilatus 6M detector and an MD3 EMBL diffractometer. Two X-ray datasets collected on a single crystal of the hNetrin-1/hDraxin-22 complex were merged using MOSFLM (Battye et al., 2011) and scaled using SCALA (Evans, 2006), resulting in a dataset with a resolution of 3.07 Å. The structure was solved by molecular replacement with PHASER (Bunkóczi et al., 2013), and the Netrin-1 laminin domain from PDB coordinates PDB: 4URT served as a search model. Each EGF domain was placed separately by MOLREP (Vagin and Teplyakov, 2010) after the laminin domain was refined in REFMAC5 (Murshudov et al., 2011). Electron density for the glycan chains was observed near residues Asn95, Asn116 and Asn131. A fragment of hDraxin-22, spanning from residue Met225 to Pro243 was iteratively built into the density near the EGF3 domain of hNetrin-1. The structure was built in Coot (Emsley et al., 2010) and refined using REFMAC5 (Murshudov et al., 2011), with 5% randomly selected reflections used for cross-validation. The structure was refined to a final Rfactor of 23.5% (Rfree = 27.1%) (Table S2). An omit map for the hDraxin-22 peptide was generated by running zero cycles in REFMAC5, and calculating a Fo-Fc map using the FFT program from the CCP4 suite (Figure S4) (Winn et al., 2011).

#### SAXS data collection and analysis

Synchrotron radiation X-ray scattering data were collected on the EMBL P12 beamline (Blanchet et al., 2015) of the storage ring PETRA III (DESY, Hamburg) (Table S1), using a PILATUS 2M pixel detector (DECTRIS, Switzerland) and 20 frames of 0.05 s exposure time. Dilution series were measured while flowing through a temperature controlled capillary at 20°C in 20mM HEPES pH 7.4, 150mM NaCl, 5mM KCl, 1mM MgCl2, 1mM DTT and 10% glycerol, at protein concentrations of 0.3 – 1.2 mg/ml. The sample-to-detector distance was 3.1 m, covering a range of momentum transfer 0.01 ≤ s ≥ 0.46 Å^-1^ (s = 4π sinθ/λ, where 2θ is the scattering angle, and λ = 1.24 Å is the X-ray wavelength). Based on comparison of successive frames, no detectable radiation damage was observed. Data from the detector were normalized to the transmitted beam intensity, averaged, placed on absolute scale relative to water and the scattering of buffer solutions subtracted. All data manipulations were performed using the ATSAS software package (Franke et al., 2017).

The forward scattering I(0) and radius of gyration, *Rg* were determined from Guinier analysis, assuming that at very small angles (s ≤ 1.3/*Rg*) the intensity is represented as *I(s) = I(0)*exp(-(*sRg*)^2^/3)). These parameters were also estimated from the full scattering curves using the indirect Fourier transform method implemented in the program GNOM (Svergun, 1992), along with the distance distribution function *p(r)* and the maximum particle dimensions *Dmax*. Molecular masses (MMs) of solutes were estimated from SAXS data by comparing the extrapolated forward scattering with that of a reference solution of bovine serum albumin, the hydrated-particle/Porod volume *V*_*p*_, where molecular mass is estimated as 0.588 times (*V*_*p*_,) and from the excluded solvent volume (*V*_*ex*_) obtained from *ab initio* modeling in the program DAMMIF (Franke and Svergun, 2009). Ensemble analysis of hDraxin was conducted using the program EOM (Tria et al., 2015) using the c-terminal domain of rDraxin (rDraxin-C, this work) as a fixed rigid body and the remaining sequence unconstrained. Theoretical scattering profiles for compact and folded hDraxin, and for unfolded hDraxin were generated using the Guinier and Debye functions as implemented in the SASVIEW software package (http://www.sasview.org/), respectively. The radius of gyration for folded and unfolded hDraxin was calculated from the Flory relation, *Rg* = R_0_N^*v*^ (Flory, 1953) for an amino acid sequence of length N = 326. R_0_ and *V* for folded and unfolded hDraxin were 3.3 and 0.34, and 1.927 and 0.598, respectively.

#### AVEXIS assays

A construct of codon optimized human Netrin-1 (VI+V) (with the pregnancy-specific glycoprotein-1 secretion signal peptide; Finci et al., 2014) and full length human Draxin (Gao et al., 2015) were cloned into AVEXIS prey and bait vectors (Addgene plasmid #36148, #36149). A mutagenesis kit (Agilent Techologies 200521) was used to produce Draxin mutants. All recombinant proteins were expressed in HEK293F (Thermo Fisher R79007) cells via PEI mediated transient transfection. The recombinant hNetrin-1 protein was harvested 3 days post transfection, hDraxin was harvested between day 3 and day 6 after transfection.

The AVEXIS assay was performed as previous described (Gao et al., 2015). Zebrafish Matrillin4 (Matn4) was used as the internal control to monitor prey protein concentrations. This protein binds to the pentamerization region (Cartiliage Oligomeric Martix Protein, COMP) (Mann et al., 2004) of the prey protein. The absorbance values were measured using a spectrophotometer (Tecan, Infinite M1000).

#### rDCC/rDraxin binding assay

HEK293T cells were transfected with full-length wild-type or mutant rDCC constructs by PEI at 50% confluence. After 24 hours, wild-type rDraxin-Flag (10 μg/ml) was added to the culture medium. After 2 hours’ incubation, the medium was removed and cells were washed 5 times by PBS and fixed for immunostaining with FLAG (Cell signaling, 3916S) antibody.

#### Statistical analysis

Statistics were done using GraphPad Prism software. Student’s t test was performed for the cell binding and AVEXIS assays. (^∗∗∗^) p < 0.001; (^∗∗^) p < 0.01; (^∗^) p < 0.05.

### Data and Software Availability

The atomic coordinates and structure factors for the crystal structures presented in this study have been deposited in the Protein Data Bank under accession numbers PDB: 5Z5K (rDraxin/DCC Ig1-Ig4 complex) and 6FKQ (hNetrin-1/hDraxin-22 complex). The accession number for the SAXS analysis of hDraxin reported in this paper is deposited in the SASBDB database: SASDBZ6.

## Supplemental Information

Document S1. Figures S1–S4 and Tables S1 and S2

Document S2. Article plus Supplemental Information
