# Supplementary material for: Structural Basis for Draxin-Modulated Axon Guidance and Fasciculation by Netrin-1 through DCC
Source: Neuron. 2018 Mar 21;97(6):1261–1267.e4. doi: 10.1016/j.neuron.2018.02.010 (PMC5871715; doi:10.1016/j.neuron.2018.02.010)
Supplement: Document S1. Figures S1–S4 and Tables S1 and S2 [file mmc1.pdf]

Neuron, Volume *97*

Supplemental Information

Structural Basis for Draxin-Modulated Axon Guidance and Fasciculation by Netrin-1 through DCC

Ying Liu, Tuhin Bhowmick, Yiqiong Liu, Xuefan Gao, Haydyn D.T. Mertens, Dmitri I. Svergun, Junyu Xiao, Yan Zhang, Jia-huai Wang, and Rob Meijers

Supplementary Table 1. SAXS Data collection and derived parameters for full-length Draxin, Related to Figure 1. Abbreviations: M_r: molecular mass; R_g: radius of gyration; D_{max}: maximal particle dimension; V_p: Porod volume; V_{ex}: Particle excluded volume.

	hDraxin
**Data collection parameters**	
Instrument	EMBL P12 (PETRA-III, Hamburg)
Beam geometry	0.2 x 0.12 mm^2
Wavelength (Å)	1.24
s range ($Å^{-1}$)[a]	0.01-0.46
Exposure time (s)	1 (20×0.05 s)
Concentration range (mg/mL)	0.3-1.2
Temperature (K)	293
**Structural parameters**[b]	
I(0) (cm^{-1}) [from *p(r)*]	0.030 ± 0.001
R_g (Å) [from *p(r)*]	44 ± 1
I(0) (cm^{-1}) (from Guinier)	0.032 ± 0.001
R_g (Å) (from Guinier)	42 ± 1
D_{max} (Å)	150
Porod volume estimate ($Å^3$)	87000 ± 10000
Excluded volume estimate ($Å^3$)	124000 ± 10000
Dry volume calculated from sequence ($Å^3$)[c]	53846[c,d]
**Molecular-mass determination**	
I(0) (cm^{-1}) Bovine serum albumin (70,000 Da)	0.050 ± 0.001
Molecular mass M_r (Da) [from *I(0)*]	44000 ± 5000
Molecular mass M_r (Da) [from Porod volume ($V_p/1.7$)]	51000 ± 5000
Molecular mass M_r (Da) [from excluded volume ($V_{ex}/2$)]	62000 ± 5000
CalculatedM_r from sequence (Da)	~44501[d]

Software employed

Primary data reduction	RADAVER
Data processing	PRIMUS/Qt
Computation of model intensities	CRYSOL

[a]Momentum transfer $s = 4\pi\sin(\theta)/\lambda$. [b]Values reported for 1.2 $mg.mL^{-1}$. [c]Dry volume determined using the server: http://www.basic.northwestern.edu/biotools/proteincalc.html. [d]Single glycosylation site not included in calculation, expected to add ~ 1.6 kDa.

Supplementary Table 2. Data collection and refinement statistics, Related to Figures 2 and 3.

	rDraxin-C/rDCC^{Ig1Ig4}	hDraxin-22 peptide/hNetrin-1
**Data collection**		
Space group	$P6_5$	$P6_422$
Cell dimensions		
a, *b*, *c* (Å)	108.07, 108.07, 130.27	130.79, 130.79 183.87
α, β, γ (°)	90, 90, 120	90, 90, 120
Resolution (Å)	2.50 (2.54–2.50)*	3.07 (3.18-3.07)
R_{pim}**	3.6 (48.5)	10.5 (67.3)
I / σI	38.3 (2.4)	8.9 (1.6)
CC1/2	1.0 (0.83)	0.99 (0.31)
Completeness (%)	99.8 (100.0)	99.9 (99.8)
Redundancy	10.9 (10.6)	32.9 (18.7)
**Refinement**		
Resolution (Å)	2.50	3.07
No. reflections	29938	17128
R_{work} / R_{free}	20.7 / 24.7	23.5/ 27.1
No. atoms		
Protein	3435	3422
Ligand	120	153
Water	34	
B-factors		
Draxin	112.9	109.7
DCC/Netrin	66.0 (rDCC^{Ig1Ig4})	75.2 (hNetrin-1)
Water	60.4	-NA-
R.m.s. deviations		
Bond lengths (Å)	0.003	0.012
Bond angles (°)	0.66	1.88
Ramachandran		
Favored (%)	94.1	89.3
Outliers (%)	0.4	0.0

*Values in parentheses are for highest-resolution shell. **Rpim (all I+ & I-)

Supplementary Figure S1. Annotated Rat Draxin sequence, Related to Figure 1

Signal peptide: MAGSVLRVPM LFLILLLFPE LYMT

Unstructured region (positively charged residues marked in blue-shaded, negatively charged residues in red font, prolines in magenta-shaded):

25

GTLASGSSARNLPETHSHLPSSALWVPQTSHHGRRGLGKKDRGPGRPRRTQE GAVVTATR

85

QASQMTPGQPPAGLLQNKELLLGLALPYPEKEARSPGWERVKKRGREHKRR RDRLRLHRG

145

RPAIRGPSSLMKKVEPSEDRMLESTMEESSTSLAPTMFFLTTADGAMPTVEES RILPVTS

205

LRPQTQPTSD

Netrin-1 binding region (in green-shaded):

218

GEVMPTLDMA LFDWTDYEDL KP

Linker (underlined glycosylation site):

244

EVWPSAKK KEKHWSHFTSDGNETSPAEG

DCC binding region (cysteines marked in yellow-shaded):

265

EPCDHHQDCLPGTCCDLREHLCTPHNRGLNNKCFDDCMCTEGLRCYAKFHR NRRVTRRKG

325

RCVEPETANGDEGSFINI

Supplementary Figure S2. Sequence alignment of Draxin, Related to Figure 1. The conserved regions are highlighted in red, showing the Draxin- 22mer peptide and Draxin-C cysteine knot domain regions are the only conserved components of Draxin. Figure prepared using ESPRIPT.

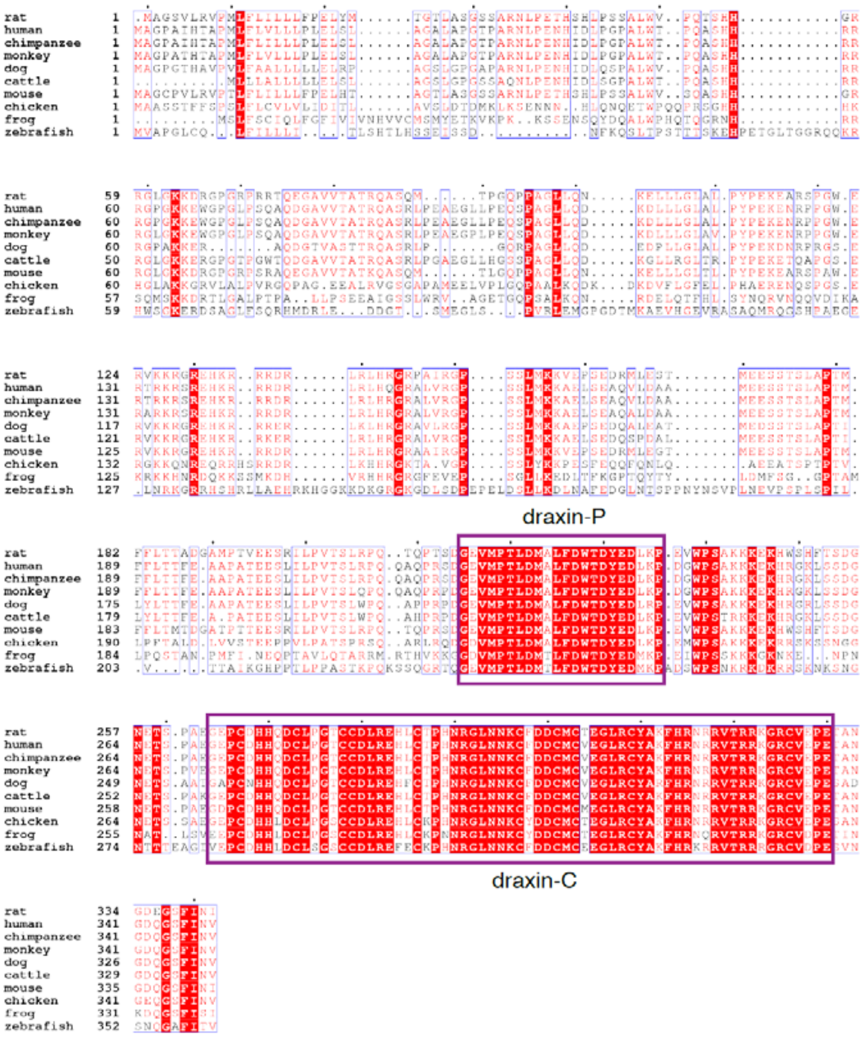

Supplementary Figure S3. Structural analysis of Draxin-C cysteine knot domain and Draxin/DCC crystal composition, Related to Figure 1. (A) SAXS ensemble analysis of rDraxin. (upper panel) Fit of the average scattering intensity of the selected ensemble of conformers from EOM (red line) to the experimental data for rDraxin (gray circles). (lower panel) Rg distance profile from EOM, the Rg distributions of random pool of conformations (black dotted line) and selected conformers (red bars) are shown. The flexibility metric R_{flex} is 82% for the selected ensemble and 85% for the pool, demonstrating the high degree of flexibility in the system. (B) SDS-PAGE gel of a dissolved rDraxin-C/rDCC co-crystal. Crystals in the condition of PEG/Ion Screen No.8 were looped out one by one, washed in 0.2 M KCl, 25% PEG 3350, and dissolved in protein sample buffer for SDS-PAGE. The SDS-PAGE was performed for 2 hours with 15% polyacrylamide to obtain the separated bands of the two components. The gel was silver stained following the manufacturer's brochure (Sigma, ProteoSilverTM Silver Stain Kit, SLBD8829). (C) Superposition and structure-based sequence alignment of rDraxin-C and Dickkopf-2 (DKK2, PDB code 2JTK). A) Ribbon diagram of rDraxin-C (magenta) and the cysteine knot domain of DKK-2 (dark cyan). B) Structure-based sequence alignment of rDraxin-C and the cysteine knot domain of DKK-2 using ESPRIPT. Cysteines are colored green, and each disulfide bond is numbered. Secondary structure is depicted as curls for alpha helices and arrows for beta strands.

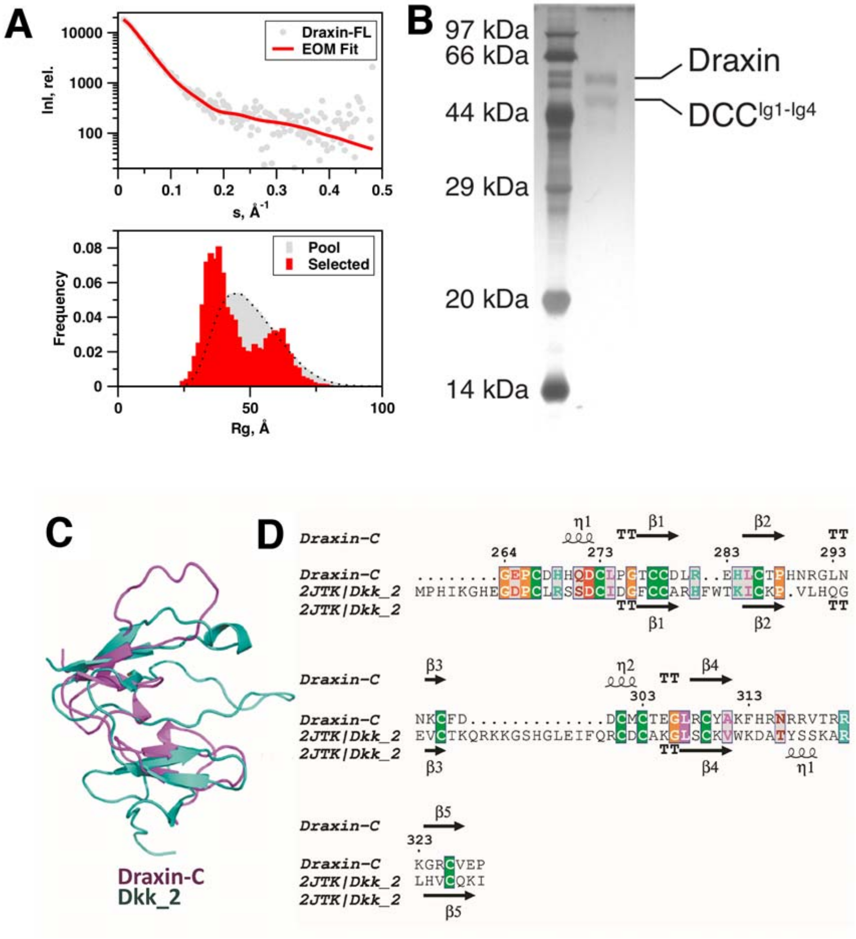
A
Draxin-FL
EOM Fit
lnI, rel.
10000
1000
100
s, Å-1
0
0.1
0.2
0.3
0.4
0.5
Pool
Selected
Frequency
0.08
0.06
0.04
0.02
0
Rg, Å
0
50
100
B
97 kDa
66 kDa
44 kDa
29 kDa
20 kDa
14 kDa
Draxin
DCCIg1-Ig4
C
Draxin-C
Dkk_2
D
Draxin-C
η1 β1 β2
TT TT
264 273 283 293
Draxin-C ........GEPCDHHQDCLPGTCCDLR..EHLCTPHNRGLN
2JTK|Dkk_2 MPHIKGHEGDPCLRSSDCIDGFCCARHFWTKICKP.VLHQG
2JTK|Dkk_2
TT TT
β1 β2
Draxin-C
β3 η2 β4
TT
303 313
Draxin-C NKCFD.............DCMCTEGIRCYAKFHRNRRVTRR
2JTK|Dkk_2 EVCTKQRKKGSHGLEIFQRCDCAKGISCKVWKDATYSSKAR
2JTK|Dkk_2
TT
β3 β4 η1
Draxin-C
β5
323
Draxin-C KGRCVEP
2JTK|Dkk_2 LHVCQKI
2JTK|Dkk_2
β5

Supplementary Figure S4. Electron density maps for the DCC/Draxin and hNetrin-1/Draxin-22 complex crystal structure, Related to Figures 2 and 3. A) Omit map (green) for the Draxin portion contoured at 3.0 sigma with the Calpha trace shown for DCC in orange, and Draxin in magenta. B). 2mFo-DFc electron density map (blue) contoured at 1 sigma including the Draxin portion. (C) Crystal structure with the electron density maps of hDraxin-22 peptide bound at site 1 (EGF 3 domain) of Netrin-1. In order to validate, maps were generated after removal of the Draxin peptide. This resulted in a strong positive density for the missing peptide chain, as displayed in Panel-I for two different orientations (location pointed by arrows). Panel-II shows the map with the hDraxin-22 peptide (shown in purple-ball and stick representation) for the corresponding orientations. The Draxin binding site of Netrin-1 is shown in orange- cartoon representation. 2mFo-DFc maps are visualized in blue (at 1 sigma level) while the Fo-Fc maps are (at 3 sigma level) in green for positive and red for negative densities.

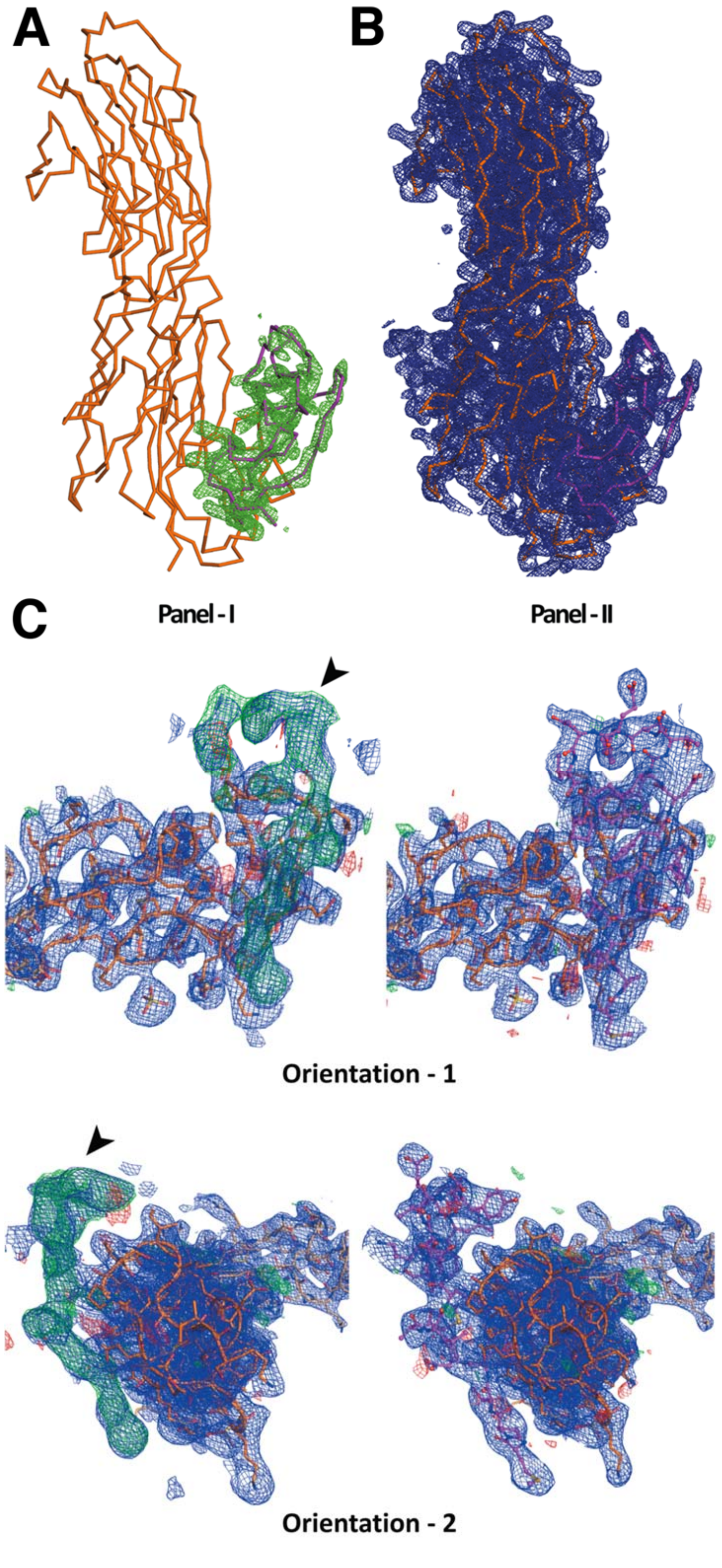
A
B
C
Panel - I
Panel - II
Orientation - 1
Orientation - 2
