# Supplementary material for: Structural Basis for Draxin-Modulated Axon Guidance and Fasciculation by Netrin-1 through DCC
Source: Neuron. 2018 Mar 21;97(6):1261–1267.e4. doi: 10.1016/j.neuron.2018.02.010 (PMC5871715; doi:10.1016/j.neuron.2018.02.010)
Supplement: Document S2. Article plus Supplemental Information [file mmc2.pdf]

Report

Neuron

Structural Basis for Draxin-Modulated Axon Guidance and Fasciculation by Netrin-1 through DCC

Highlights

- Crystal structure of cysteine knot domain of Draxin in complex with DCC
- Crystal structure of Netrin-1 in complex with a Draxin fragment
- Netrin-1 contains a competing binding site for DCC and Draxin on the EGF-3 domain
- Draxin tethers Netrin-1 and DCC together to promote fasciculation

Authors

Ying Liu, Tuhin Bhowmick, Yiqiong Liu, ..., Yan Zhang, Jia-huai Wang, Rob Meijers

Correspondence

junyuxiao@pku.edu.cn (J.X.),
yanzhang@pku.edu.cn (Y.Z.),
jwang@crystal.harvard.edu (J.-h.W.),
r.meijers@embl-hamburg.de (R.M.)

In Brief

Liu et al. report through structural investigations how Draxin associates both with Netrin-1 and its cognate receptor DCC to mediate axon guidance and fasciculation.

Liu et al., 2018, Neuron *97*, 1261–1267
March 21, 2018 © 2018 The Author(s). Published by Elsevier Inc.
https://doi.org/10.1016/j.neuron.2018.02.010

Neuron
Report

Structural Basis for Draxin-Modulated Axon Guidance and Fasciculation by Netrin-1 through DCC

Ying Liu,[1,6] Tuhin Bhowmick,[2,6] Yiqiong Liu,[1,3,6] Xuefan Gao,[2,6] Haydyn D.T. Mertens,[2] Dmitri I. Svergun,[2] Junyu Xiao,[4,*] Yan Zhang,[1,3,*] Jia-huai Wang,[1,5,*] and Rob Meijers[2,7,*]
[1]State Key Laboratory of Membrane Biology, College of Life Sciences, Peking University, Beijing 100871, China
[2]European Molecular Biology Laboratory (EMBL), Hamburg Outstation, Notkestrasse 85, D-22607 Hamburg, Germany
[3]PKU-IDG/McGovern Institute for Brain Research, Peking University, 100871 Beijing, China
[4]State Key Laboratory of Protein and Plant Gene Research, School of Life Science and Peking-Tsinghua Centre for Life Sciences, Peking University, 100871 Beijing, China
[5]Department of Medical Oncology and Department of Cancer Biology, Dana-Farber Cancer Institute, Harvard Medical School, Boston, MA 02115, USA
[6]These authors contributed equally
[7]Lead Contact
*Correspondence: junyuxiao@pku.edu.cn (J.X.), yanzhang@pku.edu.cn (Y.Z.), jwang@crystal.harvard.edu (J.-h.W.), r.meijers@embl-hamburg.de (R.M.)
https://doi.org/10.1016/j.neuron.2018.02.010

SUMMARY

Axon guidance involves the spatiotemporal interplay between guidance cues and membrane-bound cell-surface receptors, present on the growth cone of the axon. Netrin-1 is a prototypical guidance cue that binds to deleted in colorectal cancer (DCC), and it has been proposed that the guidance cue Draxin modulates this interaction. Here, we present structural snapshots of Draxin/DCC and Draxin/Netrin-1 complexes, revealing a triangular relationship that affects Netrin-mediated haptotaxis and fasciculation. Draxin interacts with DCC through the N-terminal four immunoglobulin domains, and Netrin-1 through the EGF-3 domain, in the same region where DCC binds. Netrin-1 and DCC bind to adjacent sites on Draxin, which appears to capture Netrin-1 and tether it to the DCC receptor. We propose the conformational flexibility of the single-pass membrane receptor DCC is used to promote fasciculation and regulate axon guidance through concerted Netrin-1/Draxin binding.

INTRODUCTION

The wiring of commissural neurons in the developing spinal cord is central to the development of bilateral symmetry. Commissural neurons extend axons dorsoventrally in the spinal cord that eventually cross over the midline to establish this symmetry (Chédotal, 2014). During this process, axon guidance cues, particularly the prototypical cue molecule Netrin-1, direct the growth cone situated at the tip of the axon from the roof plate toward the floor plate. Once it reaches the floor plate, the axon turns to cross the midline. Recent data appear to demonstrate that Netrin-1 derived from neural progenitors within the ventricular zone provides an adhesive axon growth substrate to guide axons through haptotaxis, and to promote axon fasciculation (Dominici et al., 2017; Varadarajan et al., 2017). Structural investigations have identified three separate receptor-binding sites on the Netrin-1 molecule at its N-terminal laminin and three following EGF domains (Finci et al., 2014; Grandin et al., 2016; Xu et al., 2014). These sites are used as a platform for Netrin-1 to engage several receptors to trigger a diverse set of signals that can determine axon navigation trajectory or cell fate. When Netrin-1 binds two DCC receptors, the homo-dimerization process has been linked to chemo-attraction of an axon (Finci et al., 2014; Kennedy et al., 1994). When DCC is co-expressed with UNC5, the Netrin-1-mediated hetero-dimerization of DCC-UNC5 turns the axon response to the opposite effect, namely chemo-repulsion (Finci et al., 2014). Furthermore, it has been shown that when a migrating cell is depleted of Netrin-1, and DCC clustering is prevented, apoptosis might ensue (Grandin et al., 2016; Krimpenfort et al., 2012; Mehlen et al., 1998).

The guidance cue Draxin (dorsal repulsive axon guidance protein) was characterized as a repulsive cue that prevents axons from misprojecting before midline crossing (Islam et al., 2009). Draxin knockout mice showed defects in fasciculation as well, indicating Draxin has an effect on axonal adhesion between pioneering and follower axons. In this context, Draxin was shown to interact with DCC present on the growth cone of the axon (Ahmed et al., 2011; Meli et al., 2015). Interestingly, a high-throughput screen for axon guidance cues and receptors present at the ventral midline revealed that Netrin-1 and Draxin bind directly to each other as well (Gao et al., 2015).

To date, it is not clear how Netrin-1 and Draxin coordinate to affect axon fasciculation and guidance. Cell binding assays indicated Draxin binds DCC at the N-terminal, membrane-distal region containing four immunoglobulin (Ig) domains (Ahmed et al., 2011). These domains form a horseshoe-shaped platform, which is common among neuronal Ig superfamily receptors (Chen et al., 2013). In contrast, Netrin-1 binds the DCC receptor on the membrane-proximal fibronectin (FN) domains FN4 and FN5-FN6, separate from where Draxin binding occurs

This is an open access article under the CC BY license (http://creativecommons.org/licenses/by/4.0/).

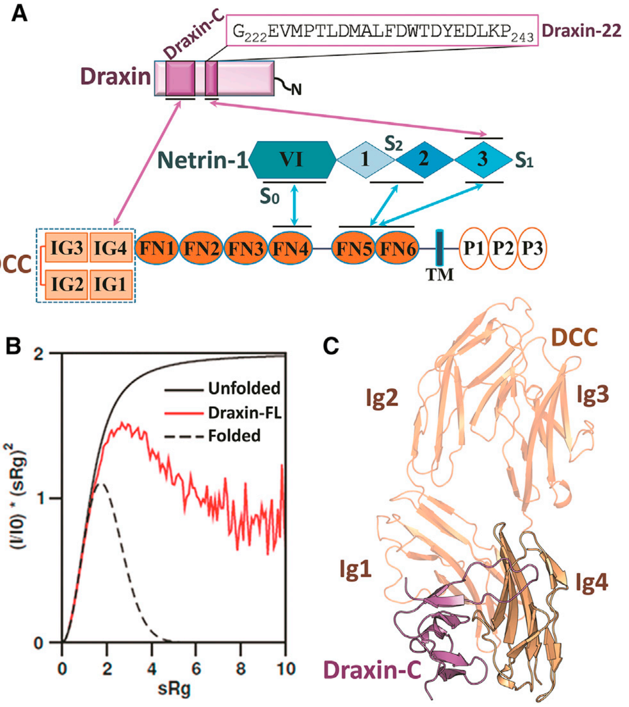

Figure 1. Overall Description of Draxin, DCC, and Netrin-1 Interactions

(A) Schematic of the interactions between the soluble guidance cues Draxin, Netrin-1, and the DCC receptor ectodomain. Draxin interacts with DCC through the C-terminal Draxin-C domain with the Ig4 domain of DCC (pink arrow). Draxin interacts with Netrin-1 through a flexible region lying next to Draxin-C, with the EGF-3 domain of Netrin-1 (pink arrow). DCC interacts with Netrin-1 through three sites (blue arrows).

(B) Dimensionless Kratky plot of the SAXS data demonstrating the partially folded nature of hDraxin in solution. Theoretical Kratky representations for compact folded (dotted black line), fully unfolded (solid black line), and the experimentally derived full-length human Draxin (solid red line) are shown.

(C) Ribbon representation of the structure of rDraxin-C in complex with DCC^{Ig1Ig4}. rDraxin-C is shown in magenta. It mainly interacts with DCC-Ig4 (beige).

(Figure 1A). Here, we present a structural characterization of Draxin itself and crystal structures of Draxin in complex with a fragment of DCC as well as with Netrin-1. We show that Draxin is largely unstructured, but that it uses a small C-terminal domain (Draxin-C) to bind DCC N-terminal Ig domains and an upstream conserved peptide motif (Draxin-22) to bind Netrin-1. Together with complementary biophysical experiments, we untangle the interactions between Draxin, Netrin-1, and DCC. We propose that Draxin facilitates Netrin-1 to act as a hub for receptors that switch between *cis* interactions involving the same axon, and *trans* interactions involving other axons or cellular substrates, to facilitate axonal adhesion and fasciculation.

RESULTS

Draxin Contains a Small Cysteine Knot Domain that Binds DCC

Draxin is predicted to consist of a signal peptide for secretion, an unstructured region covering residues 25 to 245, and a C-terminal domain (Figures 1A, S1, and S2). The folding properties of freshly purified human Draxin (hDraxin) expressed in HEK293T cells were tested by small angle X-ray scattering (SAXS; Figure 1B). The derived SAXS parameters suggest that in solution hDraxin is predominantly monomeric (Table S1). Ensemble analysis of the SAXS data (Figure S3A) shows a bi-modal size distribution, indicating that folded and unfolded states of the N-terminal part of hDraxin co-exist in solution. To identify which region of Draxin interacts with DCC, we attempted co-crystallization between full-length rat Draxin (rDraxin) and a fragment of the rat DCC receptor consisting of Ig domains 1 to 4 ($rDCC^{Ig1-Ig4}$), since Draxin binding was found to involve the N-terminal four domains of DCC (Ahmed et al., 2011).

A crystal structure was determined by molecular replacement using the known $DCC^{Ig1-Ig4}$ structure at 2.5 Å resolution (Figures 1C, S4A, and S4B). In the refined structure, a fragment of rDraxin consisting of the C-terminal region that extends from Gly264 to Pro329 can be built. The electron density for this rDraxin-C domain is continuous for residues Gly264 to Ala311 and Arg317 to Pro329 with weak density linking Ala311 to Arg317. All residues that form an interface with the DCC molecule are well defined in the electron density. When the crystals are dissolved and analyzed by SDS-PAGE, it appears the full-length rDraxin molecule is present in the crystal (Figure S3B). The solvent content for a full-length rDraxin/DCCIg1-Ig4 co-crystal is 60%, which gives enough space to accommodate the large portion of the disordered remainder of the rDraxin molecule, which is not visible in the electron density map.

In the structure of the rDraxin-C/$DCC^{Ig1-Ig4}$ complex, rDraxin-C essentially binds to the Ig4 domain of DCC. Two loops extend out from rDraxin-C, clamping at the CD loop of the Ig4 domain of DCC like a lobster grabbing her prey (Figure 2). The rDraxin-C structure consists of two sub-domains we have designated Claw1 (residues Gly264 to Asn290) and Claw2 (residues Arg291 to Pro329) of the lobster, which are kept together by a disulfide bond between Cys278 and Cys301. Each subdomain contains two finger-shaped loops that are kept together by disulfide bonds, a configuration that is typical for a cysteine knot domain. The topology of rDraxin-C is similar to the C-terminal region of the Dickkopf (DKK) protein that is involved in Wnt signaling (Cheng et al., 2011; Mao et al., 2001). Superposition of the rDraxin-C structure with DKK shows that the overall domain architectures are quite similar (Figure S3C). The disulfide bond pattern in the rDraxin-C and DKK structures is identical (Figure S3D).

When the two claws of rDraxin-C pinch the $rDCC^{Ig4}$ domain around the CD loop (Figure 2A), Claw1 clamps the CD loop at the bottom of $rDCC^{Ig4}$, whereas Claw2, a much larger loop, reaches the top of $rDCC^{Ig4}$. A total of eleven hydrogen bonds, largely arginine mediated, are formed between rDraxin-C and $rDCC^{Ig1-Ig4}$, which warrant binding specificity (Lo Conte et al., 1999). Notably, the only contacts between rDraxin-C and the $rDCC^{Ig1-Ig4}$ horseshoe outside of the Ig4 domain are two hydrogen bonds from rDraxin-C to the Ig1 domain of $rDCC^{Ig1-Ig4}$. There are also extensive hydrophobic interactions between the two binding partners to ensure binding affinity (Figure 2B). In particular, $Ile372^{DCC}$ is the most significant one,

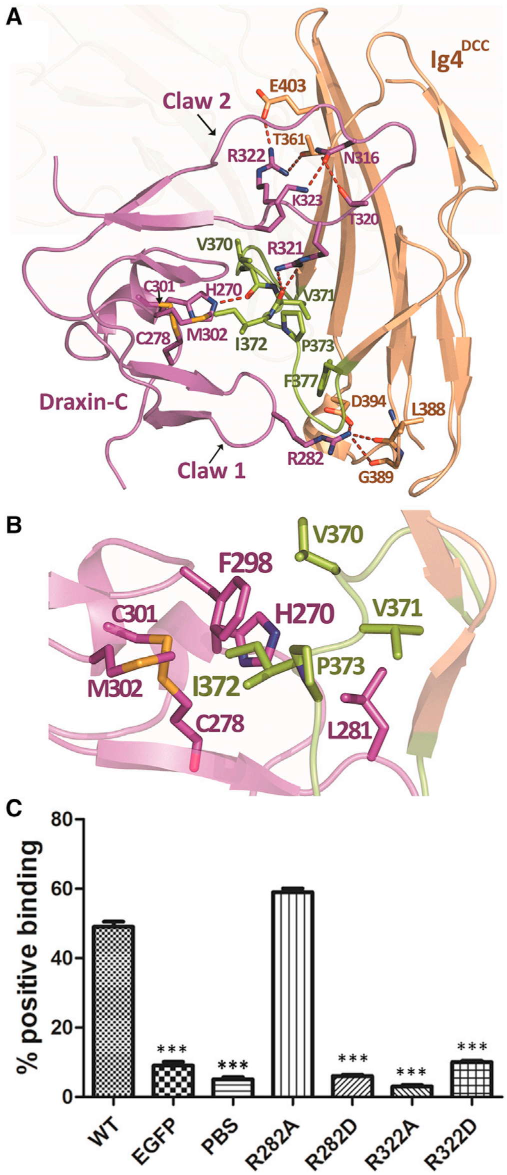

Figure 2. Characterization of Draxin/DCC Binding

(A) Overview of the interactions between rDraxin-C and the Ig4 domain of rDCC^{Ig1Ig4}. Residues mediating important interactions between the two proteins are shown as sticks and labeled. Salt bridge and hydrogen bond interactions are shown as dashed lines. The CD loop on the Ig4 domain of DCC is colored in green.

(B) Detailed view of the hydrophobic hotspot of the rDraxin-C/r DCC^{Ig1Ig4} complex. Residue Ile372 from the CD loop (colored in lemon) of rDCC-Ig4 stacks between His270 and Phe298 of rDraxin-C to form the core of the hydrophobic cluster. The core is surrounded by the hydrophobic side chains of Val370, Val371, and Pro372 from the CD loop of DCC^{Ig1Ig4} and Cys278, Leu281, Cys301, and Met302 from rDraxin-C. The view is rotated 45 degrees along the vertical axis with regard to (A).

(C) Cell-binding assays for full-length wild-type (WT) rDCC showing the percentage of DCC presenting HEK293Tcells that bind rDraxin WT and mutants Arg282Ala, Arg282Asp, Arg322Ala, and Arg322Asp. As a control, eGFP and PBS were used. Data represent mean ± SE (n = 100 for each group). One-way ANOVA, followed by a post hoc Scheffé's test, was performed. $^{**}p < 0.001$ compared with WT.

protruding out from this CD loop to be surrounded (anti-clockwise in Figure 2B) by Phe298^{Draxin}, Met302^{Draxin}, the disulfide pair Cys278^{Draxin}-Cys301^{Draxin}, His270^{Draxin}, and Leu281^{Draxin}, which might be the energetic "hot spot."

To verify the contributions of the individual residues in the binding interface between rDCC$^{Ig1\text{-}Ig4}$ and rDraxin-C, we performed mutagenesis on full-length rDraxin and tested binding to COS cells expressing full-length wild-type DCC on the cell surface (Figure 2C). The residue Arg282^{Draxin} situated on Claw1 seems to be involved in several interactions with DCC^{Ig4}. A charge-reversal mutant (Arg282Asp) abolishes binding of rDraxin to DCC, whereas an Arg282Ala mutant seems to have little effect (Figure 2C). Mutants of residue Arg322^{Draxin} present on Claw2 of rDraxin were also tested for binding to DCC presented on COS cells. Replacement of the side chain of Arg322^{Draxin} with alanine or aspartate leads to a drastic reduction in binding.

Crystal Structure of a Human Draxin/Netrin-1 Complex

Just upstream of the rDraxin-C domain (Figure S1), there is a conserved region of 22 amino acids that interacts with Netrin-1 (Figure 1A; Gao et al., 2015). A crystal structure was determined of a fragment of human Netrin-1 (hNetrin-1) consisting of the laminin and three EGF domains (residues 22 to 455) in complex with a human Draxin peptide with a length of 22 amino acids (hDraxin-22) corresponding to the region from Gly222 to Pro243 (Figures 3A and S4C).

In the hDraxin/hNetrin-1 complex, the hDraxin-22 peptide is bound to the EGF-3 domain of Netrin-1 in an extended hook-like fashion, perpendicular to the long axis of the rigid rod-like shape of the Netrin-1 molecule. The key feature of the hDraxin/hNetrin-1 complex structure is that there are two cavities on the EGF-3 domain of hNetrin-1 that are filled by the hydrophobic side chains of residues Leu228^{Draxin} and Phe233^{Draxin} of the hDraxin-22 peptide (Figure 3B). These residues are positioned to point into the cavity and are stabilized by a network of specific hydrogen bonds between the hDraxin-22 peptide and hNetrin-1 residues as well as intra-Draxin hydrogen bonds.

Previously, it was shown that Draxin interferes with Netrin-1 binding to DCC, and that this effect is localized in the 22 aa Draxin fragment (Gao et al., 2015). The structure reveals that the hDraxin-22 peptide binds hNetrin-1 at the same location as the FN5 domain of DCC. The binding epitopes overlap to a large extent (Figure 3C), surrounding the two cavities formed on the EGF-3 domain of Netrin-1. The FN5 domain of DCC employs β strands A and G to position Val848^{DCC} and Met933^{DCC} to fill the EGF-3 domain cavities of Netrin-1 (Finci et al., 2014; Xu et al., 2014). The hDraxin-22 peptide uses a hydrogen bonding network to align Phe233^{Draxin} in the same position as Val848^{DCC} from DCC, and Leu228^{Draxin} in the same position as Met933^{DCC}. It is interesting to note that in the Netrin-1/DCC structure, the Netrin-1 residue Q433^{netrin} forms two hydrogen bonds to the main chain of DCC, facilitating the hotspot binding to Val848^{DCC} and Met933^{DCC} (Finci et al., 2014). In the structure of hNetrin-1/hDraxin-22, the same Q433^{netrin} forms two hydrogen bonds to the main chain of Draxin to orient Phe233^{Draxin} and Leu228^{Draxin} for binding. The buried surface area is very similar between the Draxin/Netrin-1 interface (605 Å^2) and the Netrin-1/DCC

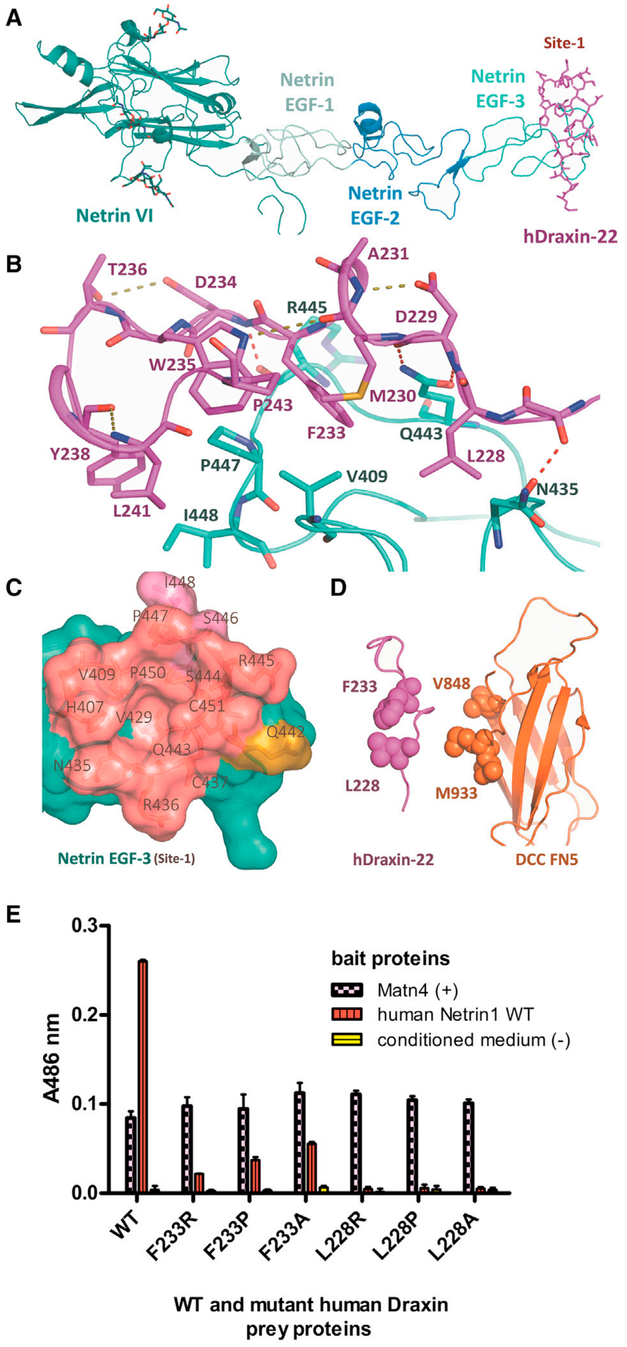

Figure 3. Crystal Structure of the hNetrin-1/hDraxin-22 Peptide

(A) An overview of the complex showing hDraxin-22 peptide (in magenta) represented as sticks binding to the Netrin-1 on the EGF-3 domain, represented in ribbon. Netrin-1 consists of the N-terminal laminin VI domain (in deep teal), followed by three EGF domains (in pale cyan, marine, and cyan).

(B) Important residues involved in hNetrin-1/hDraxin-22 interaction interface: residues Thr227, Asp229, and Asp234 of hDraxin-22 form hydrogen bonds with Asn435, Gln443, and Arg445 of hNetrin-1, respectively, while Leu228 and Phe233 of hDraxin-22 are involved in hydrophobic interactions. The hydrogen bonds between hDraxin-22 and Netrin-1 are shown in red, while intra-Draxin hydrogen bonds are shown in deep olive.

(C) A comparison between the residues of Netrin-1 involved in Draxin and DCC binding using a surface representation of Netrin-1 site 1 located at the EGF3 domain (in cyan). The residues interacting only with Draxin are colored in pink, only with DCC are colored in olive, and with both Draxin and DCC are colored in deep salmon. These residues form a hydrophobic cavity at site 1, surrounding residue Val429 of Netrin.

(D) Comparison of the crucial residues involved in Netrin-1 binding for Draxin (Leu228 and Phe233) and DCC (Met933 and Val848).

(E) AVEXIS binding results for Netrin/WT and mutant Draxin interactions. Human Netrin-1 WT bait protein (VI+V) interacts with Draxin full-length prey proteins with indicated mutations. Matn-4 bait used as the internal positive control; conditioned medium as the negative control. The A486 nm values correspond to the average of three repeats, error bars represent mean ± SD, and results were confirmed in two independent experiments.

interface (609 Å^2). Similarly, the surface complementarity values between the Draxin/Netrin-1 interface (0.67) and the Netrin-1/DCC interface (0.66) are comparable. Since there does not seem to occur any refolding of the hDraxin-22 peptide upon binding to Netrin-1, we speculate that solvation-related energy effects are the main determinant in the relatively strong binding of Draxin to Netrin-1 (Kd = 10 nM; Gao et al., 2015) compared to DCC (estimated Kd for the Fn5 domain alone is 5 μM; Xu et al., 2014).

Binding Assays Confirm Draxin Binding to Netrin-1 Occurs Only through the EGF-3 Domain

To validate the contribution of the individual Draxin residues to Netrin-1 binding, AVEXIS assays were performed (Bushell et al., 2008) with full-length hDraxin (Gao et al., 2015). Based on the crystal structure presented here, point mutations were introduced in the region of Draxin that interacts with Netrin-1. Mutagenesis of Phe233^{Draxin} to alanine leads to a reduction in Netrin-1 binding, whereas a Phe233Arg^{Draxin} mutant completely abolishes binding (Figure 3E). Importantly, mutagenesis of Leu228^{Draxin} has an even stronger effect because the Leu228Ala^{Draxin} mutant is sufficient to disrupt interaction with Netrin-1. This confirms that the residue Leu228^{Draxin}, which fills the pocket at the EGF3 domain of Netrin-1, is most crucial for binding, just like Met933^{DCC}, which occupies the same pocket in the Netrin-1/DCC interaction. The observation that single point mutants within the region of Draxin identified to bind the EGF-3 domain of Netrin-1 in the crystal structure can disrupt binding between Draxin and Netrin-1 confirms that this is the only mode of interaction between these guidance cues, despite the intrinsically disordered properties of Draxin.

DISCUSSION

The crossing of the midline by commissural axons in the developing spinal cord of vertebrae involves an intricate combination of guidance cues, including Draxin and Netrin-1 (Dudanova and Klein, 2013). Although Draxin was identified as a repulsive guidance cue based on its *in vitro* activities, the main phenotype observed in Draxin knockout mice is defasciculation. Draxin seems to facilitate the bundling of axons together, and its absence leads to the straying of individual commissural axons. Here we provide structural evidence that Draxin interacts both with Netrin-1 and DCC. Draxin binds

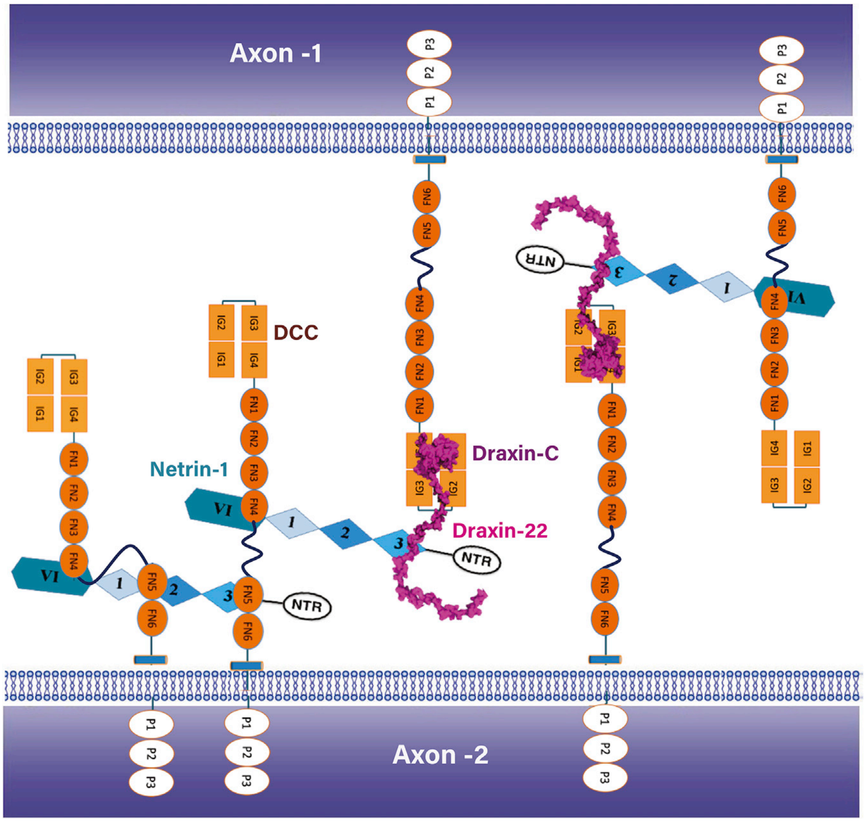

Figure 4. Proposed Model for hNetrin-1- and Draxin-Mediated Adhesion between Axons through DCC

Two opposing axons decorated with DCC (orange, individual Ig domains depicted as ovals and fibronectin domains as boxes). hNetrin-1 (different shades of blue) binds DCC at the laminin (VI) domain as well as the EGF domains (sites 1 and 2). Draxin (purple) competes with DCC to bind hNetrin-1 at site 1 and crosslinks hNetrin-1 with a DCC molecule on the opposite axon.

to the horseshoe-shaped N-terminal Ig domains of DCC, distant from the cell membrane. The crystal structure of the C-terminal region of Draxin in complex with rDCC$^{Ig1\text{-}Ig4}$ reveals a relatively weak binding site, which is verified with structure-derived mutants of Draxin in cell-based binding studies. Draxin also contains a Netrin-1 binding site, just 20 amino acids N-terminal to Draxin-C, the DCC binding domain. It covers a 22-residue region that is evolutionary conserved, but is intrinsically unstructured. The crystal structure of hNetrin-1 in complex with an hDraxin-22 peptide illustrates how Draxin binds to Netrin-1. Strikingly, Draxin binds to Netrin-1 on the EGF-3 domain of Netrin-1, involving the same region that constitutes binding site 1 for DCC. Draxin outcompetes DCC for binding to Netrin-1 on site 1, based on kinetics experiments (Gao et al., 2015; Xu et al., 2014). From the binding configurations between Draxin, DCC, and Netrin-1, it is possible to suggest a model for how Draxin promotes axon fasciculation (Figure 4). The close proximity of the DCC and Netrin-1 binding sites on Draxin enables Draxin to capture Netrin-1 molecules on the tip of DCC, away from the cell membrane of the axon. The relatively strong Draxin/Netrin-1 complex can build a bridge between two axons decorated with DCC, initiating adhesion and therefore fasciculation between the axons.

The classical model for Netrin/DCC clustering suggests that two DCC receptor ectodomains are paired by a Netrin-1 molecule, triggering the dimerization of the cytosolic domains of DCC (Finci et al., 2014; Stein et al., 2001). This dimerization will then lead to the formation of a supramolecular complex around the cytosolic domains of DCC (Lai Wing Sun et al., 2011). Surprisingly, structural studies on Netrin/DCC revealed three separate binding sites for DCC on Netrin-1 (Finci et al., 2015; Xu et al., 2014), which indicates that the pairing of DCC and other Netrin receptors is more complex. One of the binding sites (site 2) can alternatively bind DCC and UNC5, which leads to a switch from chemo-attraction to chemo-repulsion (Finci et al., 2015; Grandin et al., 2016). Now we show that another binding site (site 1) can alternatively bind DCC and Draxin. The multivalent interactions between Netrin-1 and DCC, modulated by other receptors and soluble factors such as Draxin, may be involved in the movement of the axon along a substrate-bound Netrin-1 gradient (Dominici et al., 2017; Varadarajan et al., 2017), implying that axon guidance is a more complex process than was previously thought. The exploration of the mechanistic principals underlying this key neuronal developmental process seems to enter an exciting phase.

The structural details presented here may provide general insights into the mode of action of neuronal receptors containing Ig and fibronectin domains that act as beads on a string. For instance, it was recently shown that DSCAM interacts with guidance cues Slit and Netrin-1 (Dascenco et al., 2015; Liu et al., 2009). Similar to Draxin/DCC, Slit binds to the horseshoe-shaped N-terminal region of DSCAM (Dascenco et al., 2015). As these neuronal receptors show great conformational flexibility, they allow different guidance cues that are bound to different regions of the receptor to engage each other. This may lead to an intricate network of interactions that are concentration dependent in a spatiotemporal fashion, governed by the gradients of the guidance cues. In parallel, associate guidance cues like Draxin and Dickkopf may act as recruiters that link different guidance cue systems together. In fact, Draxin has been shown to bind to LRP6, a receptor in the canonical Wnt pathway (Miyake et al., 2009). Draxin could therefore link Netrin-1-mediated dorsal-ventral axon guidance with Wnt-mediated anterior-posterior axon guidance. Further investigations into these molecular handshakes

will reveal further complexities in axon guidance and cell migration.

STAR★METHODS

Detailed methods are provided in the online version of this paper and include the following:

- KEY RESOURCES TABLE
- CONTACT FOR REAGENT AND RESOURCE SHARING
- METHOD DETAILS
  - Protein production and purification
  - Crystallization and structure determination
  - SAXS data collection and analysis
  - AVEXIS assays
  - rDCC/rDraxin binding assay
  - Statistical analysis
- DATA AND SOFTWARE AVAILABILITY

SUPPLEMENTAL INFORMATION

Supplemental Information includes four figures and two tables and can be found with this article online at https://doi.org/10.1016/j.neuron.2018.02.010.

A video abstract is available at https://doi.org/10.1016/j.neuron.2018.02.010#mmc3.

ACKNOWLEDGMENTS

The authors would like to thank Lorenzo Finci for the help with X-ray data collection on rDraxin/rDCC$^{Ig1-Ig4}$ crystals, and Anna Prudnikova and Alexandra Koetter for help in the preparation of the hNetrin-1/hDraxin-22 complex. We also thank Lisa Goodrich, Rob Smock, and Michel Koch for comments on the manuscript, and the Sample Preparation and Characterization facility (SPC) facility of EMBL at PETRA3 (DESY, Hamburg) for assistance. We are grateful to staff members of the Shanghai Synchrotron Radiation Facility (beamline BL17U) for assistance in X-ray data collection for rDraxin/rDCC$^{Ig1-Ig4}$ and the staff of EMBL Hamburg at the PETRA3 beamline P14 for the hNetrin-1/hDraxin-22 data collection. T.B. and X.G. were supported by the EMBL Interdisciplinary Postdoc Program (EIPOD) under Marie Curie COFUND actions. This work was supported by an NIH grant (HL103526) and funds from the Peking-Tsinghua Center for Life Sciences to J.-h.W, NSFC Major Research Grant (31630028 and 91632305) and the National Science Foundation of China (NSFC) Fund for Distinguished Young Scholars (81425009) to Y.Z., and the National Science Foundation of China (31570735) and the National Key Research and Development Program of China (2017YFA0505200 and 2016YFC0906000) to J.X.

AUTHOR CONTRIBUTIONS

J.-h.W. and R.M. conceived and directed the project. Ying Liu prepared Draxin and DCC and co-crystallized rDraxin/rDCC$^{Ig1-Ig4}$. T.B. prepared and crystallized hNetrin-1/hDraxin-22 and solved the structure. Yiqiong Liu performed binding assays; X.G. performed AVEXIS experiments; H.D.T.M., T.B., and D.I.S. collected and analyzed SAXS data; and J.X. solved the rDraxin/rDCC$^{Ig1-Ig4}$ structure. T.B., J.X., Y.Z., J.-h.W., and R.M. analyzed the data and wrote the manuscript.

DECLARATION OF INTERESTS

The authors declare no competing interests.

Received: October 12, 2016
Revised: October 19, 2017
Accepted: February 5, 2018
Published: March 1, 2018

REFERENCES

Afonine, P.V., Grosse-Kunstleve, R.W., Echols, N., Headd, J.J., Moriarty, N.W., Mustyakimov, M., Terwilliger, T.C., Urzhumtsev, A., Zwart, P.H., and Adams, P.D. (2012). Towards automated crystallographic structure refinement with phenix.refine. Acta Crystallogr. D Biol. Crystallogr. *68*, 352–367.

Ahmed, G., Shinmyo, Y., Ohta, K., Islam, S.M., Hossain, M., Naser, I.B., Riyadh, M.A., Su, Y., Zhang, S., Tessier-Lavigne, M., and Tanaka, H. (2011). Draxin inhibits axonal outgrowth through the netrin receptor DCC. J. Neurosci. *31*, 14018–14023.

Battye, T.G.G., Kontogiannis, L., Johnson, O., Powell, H.R., and Leslie, A.G.W. (2011). iMOSFLM: a new graphical interface for diffraction-image processing with MOSFLM. Acta Crystallogr. D Biol. Crystallogr. *67*, 271–281.

Blanchet, C.E., Spilotros, A., Schwemmer, F., Graewert, M.A., Kikhney, A., Jeffries, C.M., Franke, D., Mark, D., Zengerle, R., Cipriani, F., et al. (2015). Versatile sample environments and automation for biological solution X-ray scattering experiments at the P12 beamline (PETRA III, DESY). J. Appl. Cryst. *48*, 431–443.

Bunkóczi, G., Echols, N., McCoy, A.J., Oeffner, R.D., Adams, P.D., and Read, R.J. (2013). Phaser.MRage: automated molecular replacement. Acta Crystallogr. D Biol. Crystallogr. *69*, 2276–2286.

Bushell, K.M., Söllner, C., Schuster-Boeckler, B., Bateman, A., and Wright, G.J. (2008). Large-scale screening for novel low-affinity extracellular protein interactions. Genome Res. *18*, 622–630.

Chédotal, A. (2014). Development and plasticity of commissural circuits: from locomotion to brain repair. Trends Neurosci. *37*, 551–562.

Chen, V.B., Arendall, W.B., 3rd, Headd, J.J., Keedy, D.A., Immormino, R.M., Kapral, G.J., Murray, L.W., Richardson, J.S., and Richardson, D.C. (2010). MolProbity: all-atom structure validation for macromolecular crystallography. Acta Crystallogr. D Biol. Crystallogr. *66*, 12–21.

Chen, Q., Sun, X., Zhou, X.H., Liu, J.H., Wu, J., Zhang, Y., and Wang, J.H. (2013). N-terminal horseshoe conformation of DCC is functionally required for axon guidance and might be shared by other neural receptors. J. Cell Sci. *126*, 186–195.

Cheng, Z., Biechele, T., Wei, Z., Morrone, S., Moon, R.T., Wang, L., and Xu, W. (2011). Crystal structures of the extracellular domain of LRP6 and its complex with DKK1. Nat. Struct. Mol. Biol. *18*, 1204–1210.

Dascenco, D., Erfurth, M.-L., Izadifar, A., Song, M., Sachse, S., Bortnick, R., Urwyler, O., Petrovic, M., Ayaz, D., He, H., et al. (2015). Slit and receptor tyrosine phosphatase 69D confer spatial specificity to axon branching via Dscam1. Cell *162*, 1140–1154.

Dominici, C., Moreno-Bravo, J.A., Puiggros, S.R., Rappeneau, Q., Rama, N., Vieugue, P., Bernet, A., Mehlen, P., and Chédotal, A. (2017). Floor-plate-derived netrin-1 is dispensable for commissural axon guidance. Nature *545*, 350–354.

Dudanova, I., and Klein, R. (2013). Integration of guidance cues: parallel signaling and crosstalk. Trends Neurosci. *36*, 295–304.

Emsley, P., Lohkamp, B., Scott, W.G., and Cowtan, K. (2010). Features and development of Coot. Acta Crystallogr. D Biol. Crystallogr. *66*, 486–501.

Evans, P. (2006). Scaling and assessment of data quality. Acta Crystallogr. D Biol. Crystallogr. *62*, 72–82.

Finci, L.I., Krüger, N., Sun, X., Zhang, J., Chegkazi, M., Wu, Y., Schenk, G., Mertens, H.D.T., Svergun, D.I., Zhang, Y., et al. (2014). The crystal structure of netrin-1 in complex with DCC reveals the bifunctionality of netrin-1 as a guidance cue. Neuron *83*, 839–849.

Finci, L., Zhang, Y., Meijers, R., and Wang, J.-H. (2015). Signaling mechanism of the netrin-1 receptor DCC in axon guidance. Prog. Biophys. Mol. Biol. *118*, 153–160.

Flory, P. (1953). Principles of Polymer Chemistry (Cornell University Press).

Franke, D., and Svergun, D.I. (2009). DAMMIF, a program for rapid ab-initio shape determination in small-angle scattering. J. Appl. Cryst. *42*, 342–346.

Franke, D., Kikhney, A.G., and Svergun, D.I. (2012). Automated acquisition and analysis of small angle X-ray scattering data. Nucl. Instrum. Methods Phys. Res. A *689*, 52–59.

Franke, D., Petoukhov, M.V., Konarev, P.V., Panjkovich, A., Tuukkanen, A., Mertens, H.D.T., Kikhney, A.G., Hajizadeh, N.R., Franklin, J.M., Jeffries, C.M., and Svergun, D.I. (2017). ATSAS 2.8: a comprehensive data analysis suite for small-angle scattering from macromolecular solutions. J. Appl. Cryst. *50*, 1212–1225.

Gao, X., Metzger, U., Panza, P., Mahalwar, P., Alsheimer, S., Geiger, H., Maischein, H.-M., Levesque, M.P., Templin, M., and Söllner, C. (2015). A floor-plate extracellular protein-protein interaction screen identifies Draxin as a secreted netrin-1 antagonist. Cell Rep. *12*, 694–708.

Grandin, M., Meier, M., Delcros, J.G., Nikodemus, D., Reuten, R., Patel, T.R., Goldschneider, D., Orriss, G., Krahn, N., Boussouar, A., et al. (2016). Structural decoding of the netrin-1/UNC5 interaction and its therapeutical implications in cancers. Cancer Cell *29*, 173–185.

Islam, S.M., Shinmyo, Y., Okafuji, T., Su, Y., Naser, I.B., Ahmed, G., Zhang, S., Chen, S., Ohta, K., Kiyonari, H., et al. (2009). Draxin, a repulsive guidance protein for spinal cord and forebrain commissures. Science *323*, 388–393.

Kennedy, T.E., Serafini, T., de la Torre, J.R., and Tessier-Lavigne, M. (1994). Netrins are diffusible chemotropic factors for commissural axons in the embryonic spinal cord. Cell *78*, 425–435.

Krimpenfort, P., Song, J.-Y., Proost, N., Zevenhoven, J., Jonkers, J., and Berns, A. (2012). Deleted in colorectal carcinoma suppresses metastasis in p53-deficient mammary tumours. Nature *482*, 538–541.

Lai Wing Sun, K., Correia, J.P., and Kennedy, T.E. (2011). Netrins: versatile extracellular cues with diverse functions. Development *138*, 2153–2169.

Liu, G., Li, W., Wang, L., Kar, A., Guan, K.-L., Rao, Y., and Wu, J.Y. (2009). DSCAM functions as a netrin receptor in commissural axon pathfinding. Proc. Natl. Acad. Sci. USA *106*, 2951–2956.

Lo Conte, L., Chothia, C., and Janin, J. (1999). The atomic structure of protein-protein recognition sites. J. Mol. Biol. *285*, 2177–2198.

Mann, H.H., Ozbek, S., Engel, J., Paulsson, M., and Wagener, R. (2004). Interactions between the cartilage oligomeric matrix protein and matrilins. Implications for matrix assembly and the pathogenesis of chondrodysplasias. J. Biol. Chem. *279*, 25294–25298.

Mao, B., Wu, W., Li, Y., Hoppe, D., Stannek, P., Glinka, A., and Niehrs, C. (2001). LDL-receptor-related protein 6 is a receptor for Dickkopf proteins. Nature *411*, 321–325.

Mehlen, P., Rabizadeh, S., Snipas, S.J., Assa-Munt, N., Salvesen, G.S., and Bredesen, D.E. (1998). The DCC gene product induces apoptosis by a mechanism requiring receptor proteolysis. Nature *395*, 801–804.

Meli, R., Weisová, P., and Propst, F. (2015). Repulsive axon guidance by Draxin is mediated by protein Kinase B (Akt), glycogen synthase kinase-3β (GSK-3β) and microtubule-associated protein 1B. PLoS ONE *10*, e0119524.

Miyake, A., Takahashi, Y., Miwa, H., Shimada, A., Konishi, M., and Itoh, N. (2009). Neucrin is a novel neural-specific secreted antagonist to canonical Wnt signaling. Biochem. Biophys. Res. Commun. *390*, 1051–1055.

Murshudov, G.N., Skubák, P., Lebedev, A.A., Pannu, N.S., Steiner, R.A., Nicholls, R.A., Winn, M.D., Long, F., and Vagin, A.A. (2011). REFMAC5 for the refinement of macromolecular crystal structures. Acta Crystallogr. D Biol. Crystallogr. *67*, 355–367.

Otwinowski, Z., and Minor, W. (1997). Processing of X-ray diffraction data collected in oscillation mode. Methods Enzymol. *276*, 307–326.

Petoukhov, M.V., Franke, D., Shkumatov, A.V., Tria, G., Kikhney, A.G., Gajda, M., Gorba, C., Mertens, H.D., Konarev, P.V., and Svergun, D.I. (2012). New developments in the ATSAS program package for small-angle scattering data analysis. J. Appl. Cryst. *45*, 342–350.

Robert, X., and Gouet, P. (2014). Deciphering key features in protein structures with the new ENDscript server. Nucleic Acids Res. *42*, W320-4.

Stein, E., Zou, Y., Poo, M., and Tessier-Lavigne, M. (2001). Binding of DCC by netrin-1 to mediate axon guidance independent of adenosine A2B receptor activation. Science *291*, 1976–1982.

Svergun, D.I. (1992). Determination of the regularization parameter in indirect-transform methods using perceptual criteria. J. Appl. Cryst. *25*, 495–503.

Tria, G., Mertens, H.D.T., Kachala, M., and Svergun, D.I. (2015). Advanced ensemble modelling of flexible macromolecules using X-ray solution scattering. IUCrJ *2*, 207–217.

Vagin, A., and Teplyakov, A. (2010). Molecular replacement with MOLREP. Acta Crystallogr. D Biol. Crystallogr. *66*, 22–25.

Varadarajan, S.G., Kong, J.H., Phan, K.D., Kao, T.-J., Panaitof, S.C., Cardin, J., Eltzschig, H., Kania, A., Novitch, B.G., and Butler, S.J. (2017). Netrin1 produced by neural progenitors, not floor plate cells, is required for axon guidance in the spinal cord. Neuron *94*, 790–799.e3.

Winn, M.D., Ballard, C.C., Cowtan, K.D., Dodson, E.J., Emsley, P., Evans, P.R., Keegan, R.M., Krissinel, E.B., Leslie, A.G.W., McCoy, A., et al. (2011). Overview of the CCP4 suite and current developments. Acta Crystallogr. D Biol. Crystallogr. *67*, 235–242.

Xu, K., Wu, Z., Renier, N., Antipenko, A., Tzvetkova-Robev, D., Xu, Y., Minchenko, M., Nardi-Dei, V., Rajashankar, K.R., Himanen, J., et al. (2014). Neural migration. Structures of netrin-1 bound to two receptors provide insight into its axon guidance mechanism. Science *344*, 1275–1279.

STAR★METHODS

KEY RESOURCES TABLE

REAGENT or RESOURCE	SOURCE	IDENTIFIER
Antibodies		
penta-His primary antibody	QIAGEN	cat#34660; RRID: AB_2619735
Horse radish peroxidase-linked secondary antibody	Thermo Fisher Scientific	cat# 32230; RRID: AB_1965958
FLAG monoclonal antibody	Cell Signaling	3916S; RRID: AB_10694611
Mouse monoclonal anti rat CD4 (clone OX-68) (for AVEXIS prey and bait protein normalization)	Bio-Rad/AbD Serotec	MCA1022R; RRID: AB_567282
Chemicals, Peptides, and Recombinant Proteins		
Draxin peptide: GEVMPTLDMALFDWTDYEDLKP	Genscript	hDraxin-22
Nitrocefin CAS 41906-86-9	Merck	Cat# 484400
Deposited Data		
Crystal Structure of rDraxin/ rDCC$^{Ig1-Ig4}$ complex	This study	PDB: 5Z5K
Crystal structure of hNetrin-1/hDraxin-22 complex	This study	PDB: 6FKQ
SAXS experimental data	This study	SASBDB: SASDBZ6
Experimental Models: Cell Lines		
HEK293T cells	ATCC	CRL-11268
COS Cells	ATCC	CRL-1651
FreeStyle 293-F Cells	Thermo Fisher Scientific	R79007
Oligonucleotides		
5′ ATTTA GCGGCCGCC ATGGCAGGGTCCGTC 3′	This study	rat Draxin forward primer signal sequence.
5′ GCCCC GAGCTC GATGTTGATGAAAGATCCC 3′	This study	rat Draxin, reverse primer
5′ TGGAGCCACCCCCAGTTCGAGAAGGGCGGCT CTCTTCATTTTGTGTCTGAACC 3′	This study	rat DCC (residues 39-422) forward primer
5′ TTAAA GGTACC GGCGGCTGGAGCCACCCC CAG 3′	This study	rat DCC reverse primer
5′ AATAT GAGCTC GATGGCAGGCTTGGGG 3′	This study	rat DCC reverse primer
5′ GCGGCCGCCACC ATGGCTGGGCCTGCCATC CACACCGCTC 3′	(Gao et al., 2015)	human Draxin forward primer with signal sequence
5′ GGCGCGCC GACGTTGATGAAGGATCCCT GGTC 3′	(Gao et al., 2015)	human Draxin full length reverse primer
5′ ATAAGAAT GCGGCCGC CATGGGAACCCTC 3′	This study	common primer from pXLG to AVEXIS vector, forward primer
5′ A GGCGCGCC ATGGTGGTGGTGGTGGTG GAGCTC 3′	This study	common primer from pXLG to AVEXIS vector, reverse primer
Recombinant DNA		
Plasmid: pXLG-rDraxin	This study	N/A
Plasmid: pXLG-hNetrin-1	(Finci et al., 2014)	N/A
Plasmid: rDCC $^{Ig1-Ig4}$	(Chen et al., 2013)	N/A
Plasmid: AVEXIS-hDraxin-prey	(Gao et al., 2015)	Addgene #36148
Plasmid: AVEXIS-hDraxin-bait	(Gao et al., 2015)	Addgene #36149
Software and Algorithms		
HKL2000	(Otwinowski and Minor, 1997)	N/A
Phaser	(Bunkóczi et al., 2013)	N/A
Coot	(Emsley et al., 2010)	N/A
Phenix	(Afonine et al., 2012)	N/A
Molprobity	(Chen et al., 2010)	N/A

(*Continued on next page*)

Continued

REAGENT or RESOURCE	SOURCE	IDENTIFIER
Pymol	The PyMOL Molecular Graphics System, Version 1.7.x Schrödinger, LLC	N/A
ESPRIPT	(Robert and Gouet, 2014)	N/A
MOSFLM	(Battye et al., 2011)	N/A
SCALA	(Evans, 2006)	N/A
MOLREP	(Vagin and Teplyakov, 2010)	N/A
REFMAC5	(Murshudov et al., 2011)	N/A
CCP4 Suite	(Winn et al., 2011)	N/A
Prism	GraphPad	N/A
RADAVER	Franke et al. (2012)	https://www.embl-hamburg.de/biosaxs/download.html
PRIMUS/Qt	Petoukhov et al. (2012)	https://www.embl-hamburg.de/biosaxs/download.html
CRYSOL	Petoukhov et al. (2012)	https://www.embl-hamburg.de/biosaxs/download.html
DAMMIF	Franke and Svergun, 2009	https://www.embl-hamburg.de/biosaxs/download.html
SASVIEW	NSF DANSE	http://www.sasview.org
Other		
Mutagenesis kit	Agilent Techologies	#200521
Dulbecco's modified eagle medium	DMEM, Biochrom	cat# F 0435
L-glutamine	Biochrom	cat# K 0293
fetal-bovine serum	Biochrom	cat# S 0615
Protease Inhibitor Cocktail tablets	cOmplete ULTRA Tablets, Roche	05892791001
Ni-Sepharose Excel resin	GE Healthcare	cat# 17-3712-02
HiLoad 16/60 Superdex 75 column	GE Healthcare	#28-9893-33
Nickel-NTA agarose beads	QIAGEN	Cat No./ID: 30250

CONTACT FOR REAGENT AND RESOURCE SHARING

Further information and requests for reagents should be directed to and will be fulfilled by the Lead Contact, Rob Meijers (r.meijers@embl-hamburg.de).

METHOD DETAILS

Protein production and purification

The DNA fragment of full-length rDraxin was amplified from rat embryonic cDNA library, and subcloned into the pXLG vector kindly provided by David Hacker and Florian Wurm (Protein Expression Core Facility, EPFL Lausanne, Switzerland). The rat rDCC $^{Ig1-Ig4}$ construct previously described (Chen et al., 2013) was subcloned into the pXLG vector covering residues 39 to 421. The DNA fragment of full-length human Draxin (aa 1-349, GenBank: KM655686) was amplified and subcloned into modified AVEXIS bait vector (Addgene plasmid #36148) with C-terminal removal of CD4 and replaced by a purification tag (TEV-StrepII-linker-StrepII-linker-9xHis) for use in SAXS. Human Netrin-1 covering residues 39 to 457 was subcloned into pXLG as previously described (Finci et al., 2014). For rDCC $^{Ig1-Ig4}$ and hNetrin-1, the PSG1 signal peptide was used to secrete the protein and all constructs contained a C-terminal 6xHis tag. The proteins were expressed in adherent HEK293T cells (ATCC Catalog No. CRL-11268) through transient transfection in roller bottles. Cells were cultured in Dulbecco's modified eagle medium (DMEM, Biochrom cat# F 0435), containing nonessential amino acids, 2 mM L-glutamine and 0.2% fetal-bovine serum (Biochrom, cat# K 0283,cat# K 0293, cat# S 0615) at 37°C in a 5% CO_2 atmosphere in a Wheaton incubator. The expressed protein was secreted into the culture medium, which was harvested four to six days post transfection. The medium was filtered through filter paper (Whatman). Expression was confirmed by western blot analysis using a penta-His primary antibody (QIAGEN, cat#34660) and a HRP-linked secondary antibody (Pierce, cat# 32230). Protease Inhibitor Cocktail tablets [cOmplete ULTRA Tablets, Mini, EDTA-free, EASYpack (05892791001 Roche)] were added to the medium (2 tablets per liter) immediately after harvesting.

Both rDraxin and rDCC $^{Ig1-Ig4}$ containing media were dialyzed in 20 mMTris-HCl, pH7.5, 200mMNaCl. These proteins were affinity purified by Ni column (QIAGEN Ni-NTA agarose), followed by size exclusion chromatography using a HiLoad 16/60 Superdex 200 column from GE Healthcare. Harvested media containing recombinant hNetrin-1 was then incubated at 4°C with 1.5 ml Ni-Sepharose Excel resin (GE Healthcare, cat# 17-3712-02) per liter medium, overnight with slow stirring. hNetrin-1 was eluted in 1ml fractions with 20 mM sodium phosphate, 500 mM NaCl, 500 mM imidazole at pH 7.4. The total elution volume collected was 5ml from 2 l of conditioned media. Next, the eluent from the affinity chromatography was subjected to size exclusion chromatography using a HiLoad 16/60 Superdex 75 prep grade column equilibrated in 50 mM MES buffer pH 6.0, containing 250 mM NaCl and 1 mM DTT, with a flow rate of 1 mL per minute. The hNetrin-1 peak from the size exclusion step was collected in 0.5 mL fractions in a deep well block. After inspection through SDS-PAGE gel, fractions containing hNetrin-1 with > 90% purity were combined and concentrated using an Amicon Ultra-4 Centrifugal Filter (Millipore, cat# UFC800324) to 1mg/ml (~20 uM).

Crystallization and structure determination

Purified rDraxin and rDCC$^{Ig1-Ig4}$ were combined in 1.2:1 molar ratio, and incubated at 4°C for 2 hours before loading onto the size exclusion column. The complex peak fractions were collected and concentrated to ~12mg/ml for crystallization. Crystals appeared readily at a few conditions, the best of which is in the No.8 condition of PEG/Ion Screen (HR2-126 Hampton). It was optimized to 0.2 M KCl, 3% PEG 3350 to get well-diffracting crystals. Diffraction data were collected at SSRF (Shanghai Synchrotron Radiation Facility, China) beamline BL17U and processed with HKL2000 (HKL Research) (Otwinowski and Minor, 1997). The structure was determined by molecular replacement with Phaser (Bunkóczi et al., 2013) using the DCC$^{Ig1-Ig4}$ structure (PDB: 3LAF) as the search model. The structure was built in Coot (Emsley et al., 2010) and refined using Phenix (Afonine et al., 2012), with 5% randomly selected reflections used for cross-validation. The final working R and free R factors were 20.7% and 24.7%, respectively (Table S2). The stereochemistry was checked with Molprobity (Chen et al., 2010), indicating good overall geometry with only 0.4% of the residues in disallowed regions of the Ramachandran plot. Structure diagrams were prepared with Pymol (The PyMOL Molecular Graphics System, Version 1.7.x Schrödinger, LLC). Sequence alignments were prepared with ESPRIPT (Robert and Gouet, 2014).

A 22-mer peptide (sequence: GEVMPTLDMALFDWTDYEDLKP) corresponding to the residue spanning 222-243 of human Draxin (UniProtKB entry: Q8NBI3 DRAXI_HUMAN) was ordered from Genscript. The purity of the synthesized peptide was > 75%. A peptide stock of 1mM was prepared by dissolving the peptide in water, first by lowering the pH to 5.5 and then gradually bringing it back to neutrality.

hNetrin-1 and hDraxin-22 peptide were mixed at 1:1.5 molar ratio and incubated for an hour prior to concentration to 6.5 mg/mL. Crystals were obtained in two conditions containing 1.6 M ammonium sulfate and 0.1 M Sodium citrate, at pH 4 and pH 5, respectively. Crystals were cryo-protected in 0.1M Sodium citrate (pH 4.0 or pH 5.0, based on the crystallization condition), 1.6M ammonium sulfate, 15% (v/v) Ethylene glycol prior to flash-cooling to 100K. X-ray diffraction data were collected on the P14 beamline of EMBL Hamburg situated at the PETRA3 synchrotron. The beamline was equipped with a Pilatus 6M detector and an MD3 EMBL diffractometer. Two X-ray datasets collected on a single crystal of the hNetrin-1/hDraxin-22 complex were merged using MOSFLM (Battye et al., 2011) and scaled using SCALA (Evans, 2006), resulting in a dataset with a resolution of 3.07 Å. The structure was solved by molecular replacement with PHASER (Bunkóczi et al., 2013), and the Netrin-1 laminin domain from PDB coordinates PDB: 4URT served as a search model. Each EGF domain was placed separately by MOLREP (Vagin and Teplyakov, 2010) after the laminin domain was refined in REFMAC5 (Murshudov et al., 2011). Electron density for the glycan chains was observed near residues Asn95, Asn116 and Asn131. A fragment of hDraxin-22, spanning from residue Met225 to Pro243 was iteratively built into the density near the EGF3 domain of hNetrin-1. The structure was built in Coot (Emsley et al., 2010) and refined using REFMAC5 (Murshudov et al., 2011), with 5% randomly selected reflections used for cross-validation. The structure was refined to a final Rfactor of 23.5% (Rfree = 27.1%) (Table S2). An omit map for the hDraxin-22 peptide was generated by running zero cycles in REFMAC5, and calculating a Fo-Fc map using the FFT program from the CCP4 suite (Figure S4) (Winn et al., 2011).

SAXS data collection and analysis

Synchrotron radiation X-ray scattering data were collected on the EMBL P12 beamline (Blanchet et al., 2015) of the storage ring PETRA III (DESY, Hamburg) (Table S1), using a PILATUS 2M pixel detector (DECTRIS, Switzerland) and 20 frames of 0.05 s exposure time. Dilution series were measured while flowing through a temperature controlled capillary at 20°C in 20mM HEPES pH 7.4, 150mM NaCl, 5mM KCl, 1mM MgCl2, 1mM DTT and 10% glycerol, at protein concentrations of 0.3 – 1.2 mg/ml. The sample-to-detector distance was 3.1 m, covering a range of momentum transfer $0.01 \leq s \geq 0.46$ Å^{-1} ($s = 4\pi \sin\theta/\lambda$, where 2θ is the scattering angle, and $\lambda = 1.24$ Å is the X-ray wavelength). Based on comparison of successive frames, no detectable radiation damage was observed. Data from the detector were normalized to the transmitted beam intensity, averaged, placed on absolute scale relative to water and the scattering of buffer solutions subtracted. All data manipulations were performed using the ATSAS software package (Franke et al., 2017).

The forward scattering I(0) and radius of gyration, *Rg* were determined from Guinier analysis, assuming that at very small angles ($s \leq 1.3/Rg$) the intensity is represented as $I(s) = I(0)\exp(-(sRg)^2/3)$). These parameters were also estimated from the full scattering curves using the indirect Fourier transform method implemented in the program GNOM (Svergun, 1992), along with the distance distribution function *p(r)* and the maximum particle dimensions *Dmax*. Molecular masses (MMs) of solutes were estimated from SAXS data by comparing the extrapolated forward scattering with that of a reference solution of bovine serum

albumin, the hydrated-particle/Porod volume V_p, where molecular mass is estimated as 0.588 times (V_p,) and from the excluded solvent volume (V_{ex}) obtained from *ab initio* modeling in the program DAMMIF (Franke and Svergun, 2009). Ensemble analysis of hDraxin was conducted using the program EOM (Tria et al., 2015) using the c-terminal domain of rDraxin (rDraxin-C, this work) as a fixed rigid body and the remaining sequence unconstrained. Theoretical scattering profiles for compact and folded hDraxin, and for unfolded hDraxin were generated using the Guinier and Debye functions as implemented in the SASVIEW software package (http://www.sasview.org/), respectively. The radius of gyration for folded and unfolded hDraxin was calculated from the Flory relation, $Rg = R_0N^{\nu}$ (Flory, 1953) for an amino acid sequence of length N = 326. R_0 and V for folded and unfolded hDraxin were 3.3 and 0.34, and 1.927 and 0.598, respectively.

AVEXIS assays

A construct of codon optimized human Netrin-1 (VI+V) (with the pregnancy-specific glycoprotein-1 secretion signal peptide; Finci et al., 2014) and full length human Draxin (Gao et al., 2015) were cloned into AVEXIS prey and bait vectors (Addgene plasmid #36148, #36149). A mutagenesis kit (Agilent Techologies 200521) was used to produce Draxin mutants. All recombinant proteins were expressed in HEK293F (Thermo Fisher R79007) cells via PEI mediated transient transfection. The recombinant hNetrin-1 protein was harvested 3 days post transfection, hDraxin was harvested between day 3 and day 6 after transfection.

The AVEXIS assay was performed as previous described (Gao et al., 2015). Zebrafish Matrillin4 (Matn4) was used as the internal control to monitor prey protein concentrations. This protein binds to the pentamerization region (Cartiliage Oligomeric Martix Protein, COMP) (Mann et al., 2004) of the prey protein. The absorbance values were measured using a spectrophotometer (Tecan, Infinite M1000).

rDCC/rDraxin binding assay

HEK293T cells were transfected with full-length wild-type or mutant rDCC constructs by PEI at 50% confluence. After 24 hours, wild-type rDraxin-Flag (10 μg/ml) was added to the culture medium. After 2 hours' incubation, the medium was removed and cells were washed 5 times by PBS and fixed for immunostaining with FLAG (Cell signaling, 3916S) antibody.

Statistical analysis

Statistics were done using GraphPad Prism software. Student's t test was performed for the cell binding and AVEXIS assays. (***) $p < 0.001$; (**) $p < 0.01$; (*) $p < 0.05$.

DATA AND SOFTWARE AVAILABILITY

The atomic coordinates and structure factors for the crystal structures presented in this study have been deposited in the Protein Data Bank under accession numbers PDB: 5Z5K (rDraxin/DCC Ig1-Ig4 complex) and 6FKQ (hNetrin-1/hDraxin-22 complex). The accession number for the SAXS analysis of hDraxin reported in this paper is deposited in the SASBDB database: SASDBZ6.

Neuron, Volume *97*

Supplemental Information

Structural Basis for Draxin-Modulated Axon Guidance and Fasciculation by Netrin-1 through DCC

Ying Liu, Tuhin Bhowmick, Yiqiong Liu, Xuefan Gao, Haydyn D.T. Mertens, Dmitri I. Svergun, Junyu Xiao, Yan Zhang, Jia-huai Wang, and Rob Meijers

Supplementary Table 1. SAXS Data collection and derived parameters for full-length Draxin, Related to Figure 1. Abbreviations: M_r: molecular mass; R_g: radius of gyration; D_{max}: maximal particle dimension; V_p: Porod volume; V_{ex}: Particle excluded volume.

	hDraxin
**Data collection parameters**	
Instrument	EMBL P12 (PETRA-III, Hamburg)
Beam geometry	0.2 x 0.12 mm^2
Wavelength (Å)	1.24
s range ($Å^{-1}$)[a]	0.01-0.46
Exposure time (s)	1 (20×0.05 s)
Concentration range (mg/mL)	0.3-1.2
Temperature (K)	293
**Structural parameters**[b]	
I(0) (cm^{-1}) [from *p(r)*]	0.030 ± 0.001
R_g (Å) [from *p(r)*]	44 ± 1
I(0) (cm^{-1}) (from Guinier)	0.032 ± 0.001
R_g (Å) (from Guinier)	42 ± 1
D_{max} (Å)	150
Porod volume estimate ($Å^3$)	87000 ± 10000
Excluded volume estimate ($Å^3$)	124000 ± 10000
Dry volume calculated from sequence ($Å^3$)[c]	53846[c,d]
**Molecular-mass determination**	
I(0) (cm^{-1}) Bovine serum albumin (70,000 Da)	0.050 ± 0.001
Molecular mass M_r (Da) [from *I(0)*]	44000 ± 5000
Molecular mass M_r (Da) [from Porod volume ($V_p/1.7$)]	51000 ± 5000
Molecular mass M_r (Da) [from excluded volume ($V_{ex}/2$)]	62000 ± 5000
CalculatedM_r from sequence (Da)	~44501[d]

**Software employed**	
Primary data reduction	RADAVER
Data processing	PRIMUS/Qt
Computation of model intensities	CRYSOL

[a]Momentum transfer $s = 4\pi\sin(\theta)/\lambda$. [b]Values reported for 1.2 mg.mL^{-1}.[c]Dry volume determined using the server: http://www.basic.northwestern.edu/biotools/proteincalc.html.[d]Single glycosylation site not included in calculation, expected to add ~ 1.6 kDa.

Supplementary Table 2. Data collection and refinement statistics, Related to Figures 2 and 3.

	rDraxin-C/rDCC^{Ig1Ig4}	hDraxin-22 peptide/hNetrin-1
**Data collection**		
Space group	$P6_5$	$P6_422$
Cell dimensions		
a, *b*, *c* (Å)	108.07, 108.07, 130.27	130.79, 130.79 183.87
α, β, γ (°)	90, 90, 120	90, 90, 120
Resolution (Å)	2.50 (2.54–2.50)*	3.07 (3.18-3.07)
R_{pim}**	3.6 (48.5)	10.5 (67.3)
$I / \sigma I$	38.3 (2.4)	8.9 (1.6)
CC1/2	1.0 (0.83)	0.99 (0.31)
Completeness (%)	99.8 (100.0)	99.9 (99.8)
Redundancy	10.9 (10.6)	32.9 (18.7)
**Refinement**		
Resolution (Å)	2.50	3.07
No. reflections	29938	17128
R_{work} / R_{free}	20.7 / 24.7	23.5/ 27.1
No. atoms		
Protein	3435	3422
Ligand	120	153
Water	34	
B-factors		
Draxin	112.9	109.7
DCC/Netrin	66.0 (rDCC^{Ig1Ig4})	75.2 (hNetrin-1)
Water	60.4	-NA-
R.m.s. deviations		
Bond lengths (Å)	0.003	0.012
Bond angles (°)	0.66	1.88
Ramachandran		
Favored (%)	94.1	89.3
Outliers (%)	0.4	0.0

*Values in parentheses are for highest-resolution shell. **Rpim (all I+ & I-)

Supplementary Figure S1. Annotated Rat Draxin sequence, Related to Figure 1

Signal peptide: MAGSVLRVPM LFLILLLFPE LYMT

Unstructured region (positively charged residues marked in blue-shaded, negatively charged residues in red font, prolines in magenta-shaded):

25

GTLASGSSARNLPETHSHLPSSALWVPQTSHHGRRGLGKKDRGPGRPRRTQE GAVVTATR

85

QASQMTPGQPPAGLLQNKELLLGLALPYPEKEARSPGWERVKKRGREHKRR RDRLRLHRG

145

RPAIRGPSSLMKKVEPSEDRMLESTMEESSTSLAPTMFFLTTADGAMPTVEES RILPVTS

205

LRPQTQPTSD

Netrin-1 binding region (in green-shaded):

218

GEVMPTLDMA LFDWTDYEDL KP

Linker (underlined glycosylation site):

244

EVWPSAKK KEKHWSHFTSDGNETSPAEG

DCC binding region (cysteines marked in yellow-shaded):

265

EPCDHHQDCLPGTCCDLREHLCTPHNRGLNNKCFDDCMCTEGLRCYAKFHR NRRVTRRKG

325

RCVEPETANGDEGSFINI

Supplementary Figure S2. Sequence alignment of Draxin, Related to Figure 1. The conserved regions are highlighted in red, showing the Draxin- 22mer peptide and Draxin-C cysteine knot domain regions are the only conserved components of Draxin. Figure prepared using ESPRIPT.

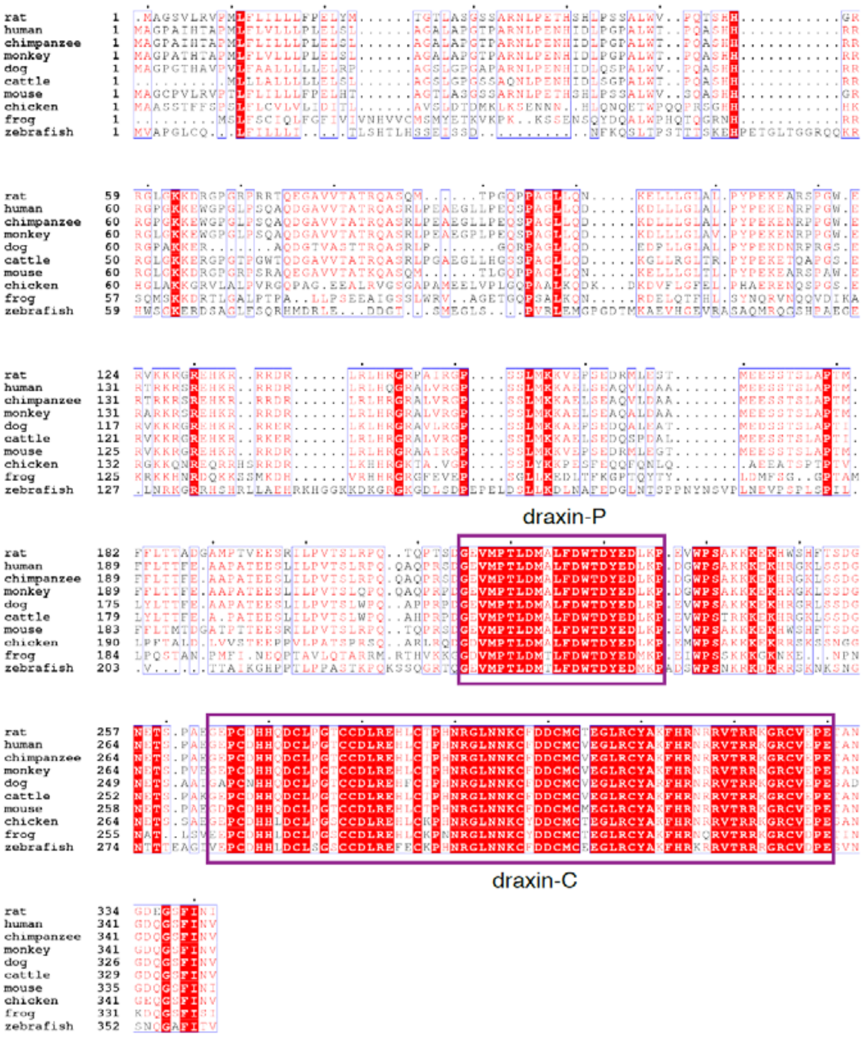

Supplementary Figure S3. Structural analysis of Draxin-C cysteine knot domain and Draxin/DCC crystal composition, Related to Figure 1. (A) SAXS ensemble analysis of rDraxin. (upper panel) Fit of the average scattering intensity of the selected ensemble of conformers from EOM (red line) to the experimental data for rDraxin (gray circles). (lower panel) Rg distance profile from EOM, the Rg distributions of random pool of conformations (black dotted line) and selected conformers (red bars) are shown. The flexibility metric R_{flex} is 82% for the selected ensemble and 85% for the pool, demonstrating the high degree of flexibility in the system. (B) SDS-PAGE gel of a dissolved rDraxin-C/rDCC co-crystal. Crystals in the condition of PEG/Ion Screen No.8 were looped out one by one, washed in 0.2 M KCl, 25% PEG 3350, and dissolved in protein sample buffer for SDS-PAGE. The SDS-PAGE was performed for 2 hours with 15% polyacrylamide to obtain the separated bands of the two components. The gel was silver stained following the manufacturer's brochure (Sigma, ProteoSilverTM Silver Stain Kit, SLBD8829). (C) Superposition and structure-based sequence alignment of rDraxin-C and Dickkopf-2 (DKK2, PDB code 2JTK). A) Ribbon diagram of rDraxin-C (magenta) and the cysteine knot domain of DKK-2 (dark cyan). B) Structure-based sequence alignment of rDraxin-C and the cysteine knot domain of DKK-2 using ESPRIPT. Cysteines are colored green, and each disulfide bond is numbered. Secondary structure is depicted as curls for alpha helices and arrows for beta strands.

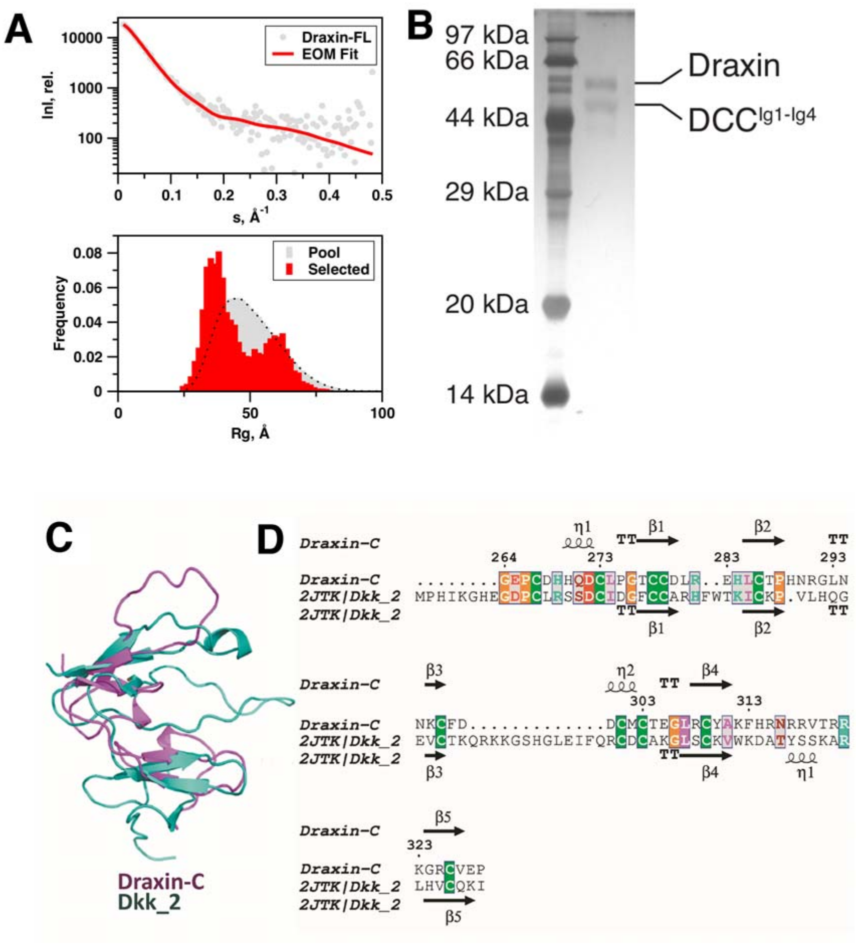
A
Draxin-FL
EOM Fit
lnI, rel.
s, Å⁻¹
Pool
Selected
Frequency
Rg, Å
B
97 kDa
66 kDa
44 kDa
29 kDa
20 kDa
14 kDa
Draxin
DCC Ig1-Ig4
C
Draxin-C
Dkk_2
D
Draxin-C
2JTK|Dkk_2
.........GEPCDHHQDCLPGTCCDLR..EHICTPHNRGLN
MPHIKGHEGDPCLRSSDCIDGFCCARHFWTKICKP.VLHQG
NKCFD.............DCMCTEGLRCYAKFHRNRRVTRR
EVCTKQRKKGSHGLEIFQRCDCAKGLSCKVWKDATYSSKAR
KGRCVEP
LHVCQKI

Supplementary Figure S4. Electron density maps for the DCC/Draxin and hNetrin-1/Draxin-22 complex crystal structure, Related to Figures 2 and 3. A) Omit map (green) for the Draxin portion contoured at 3.0 sigma with the Calpha trace shown for DCC in orange, and Draxin in magenta. B). 2mFo-DFc electron density map (blue) contoured at 1 sigma including the Draxin portion. (C) Crystal structure with the electron density maps of hDraxin-22 peptide bound at site 1 (EGF 3 domain) of Netrin-1. In order to validate, maps were generated after removal of the Draxin peptide. This resulted in a strong positive density for the missing peptide chain, as displayed in Panel-I for two different orientations (location pointed by arrows). Panel-II shows the map with the hDraxin-22 peptide (shown in purple-ball and stick representation) for the corresponding orientations. The Draxin binding site of Netrin-1 is shown in orange- cartoon representation. 2mFo-DFc maps are visualized in blue (at 1 sigma level) while the Fo-Fc maps are (at 3 sigma level) in green for positive and red for negative densities.

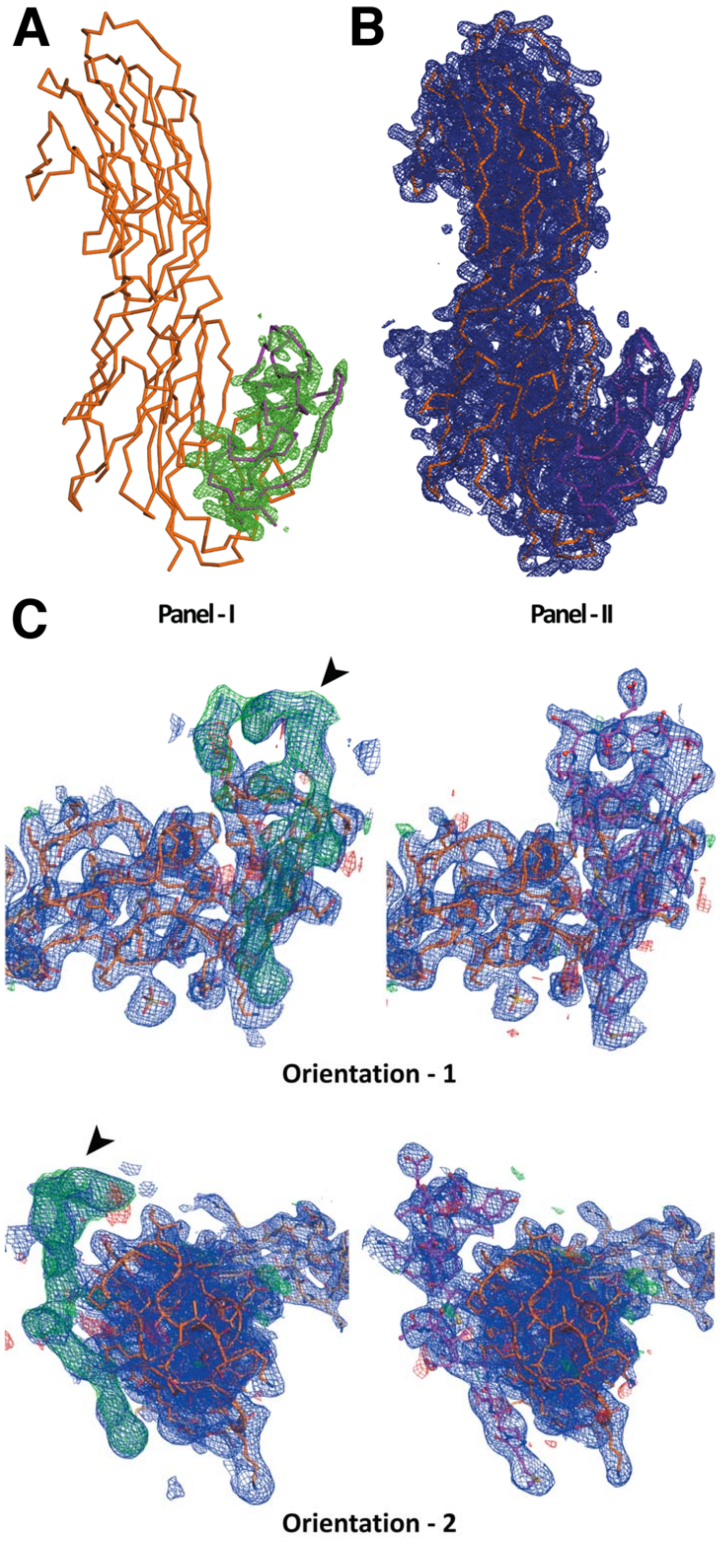
A
B
C
Panel - I
Panel - II
Orientation - 1
Orientation - 2
